# Dietary greenhouse gas emissions and resource use among Bavarian adults: associations with sociodemographics and food choices

**DOI:** 10.3389/fnut.2025.1542254

**Published:** 2025-04-09

**Authors:** Sebastian Gimpfl, Sofia Schwarz, Florian Rohm, Nadine Ohlhaut, Christine Röger, Melanie Senger, Martin Kussmann, Jakob Linseisen, Kurt Gedrich

**Affiliations:** ^1^ZIEL – Institute for Food & Health, AG Public Health Nutrition, Technical University of Munich, Freising-Weihenstephan, Germany; ^2^Chair of Epidemiology, University of Augsburg, Augsburg, Germany; ^3^Competence Center for Nutrition (KErn), Bavarian Research Institution for Agriculture (LfL), Freising, Germany; ^4^Institute for Medical Information Processing, Biometry, and Epidemiology, Medical Faculty, Ludwig-Maximilians-University of Munich, Munich, Germany

**Keywords:** greenhouse gas emissions, land use, water footprint, 3rd Bavarian Food Consumption Survey, sustainability

## Abstract

**Background:**

This study assessed dietary greenhouse gas emission (GHGE), land use (LU), and water footprint (WFP) among Bavarian residents while exploring sociodemographic characteristics, food consumption patterns, sustainability beliefs, and behaviors across GHGE quintiles.

**Methods and design:**

The 3rd Bavarian Food Consumption Survey (BVS III) was conducted from October 2021 to January 2023, involving participants aged 18–75 years. The study employed demographic weighting to represent the Bavarian population. Dietary data (*N* = 1,100) were linked to sustainability databases.

**Results:**

In Bavaria, the average dietary GHGE is 6.14 kg CO_2_eq, with LU at 7.50 m^2^*yr. and WFP at 4.39 kiloliters per 2,500 kcal. Multivariate regression analyses indicated that females had significantly higher GHGE (*β* = 0.204, *p* = 0.023) and WFP (*β* = 0.466, *p* < 0.001) compared to males. Waist circumference was positively associated with GHGE (*β* = 0.011, *p* < 0.001) and LU (*β* = 0.035, *p* < 0.001). Participants following vegetarian or vegan diets show significantly lower GHGE, LU, and WFP than omnivores. High CO_2_eq emitters also consumed more coffee, tea, and most foods of animal origin. Lowest CO_2_eq emitters are more inclined to reduce meat consumption (91% vs. 61–77%, *p* = 0.012), while higher emitters focused on purchasing regional foods (93–95% vs. 80%, *p* = 0.041).

**Conclusion:**

This study provided a view of dietary sustainability metrics in Bavaria. Considering energy-adjusted diets, higher emissions are associated with being female, having a higher waist circumference, and following an omnivorous diet. Increased consumption of animal products, coffee, and tea contributed to greater environmental impacts.

## 1 Introduction

One of the greatest challenges of the 21st century is to feed a growing world population with healthy food without exceeding planetary boundaries (1). Our diets and food systems are currently significant drivers of climate change – accounting for 34% of global greenhouse gas emissions (GHGE) (2). In Germany and overall Europe, diets have significant and growing environmental impact, exceeding a fair share of global ecological resources. This can largely be attributed to the high consumption of animal products, which are associated with a greater carbon footprint, land use (LU) and water footprint (WFP) (3–6). Recent studies have highlighted the necessity of dietary shifts toward more sustainable, less resource-intensive options to mitigate climate change and ameliorate environmental pressures (1, 7, 8). Evidence from systematic reviews showed that transitioning from current Western diets with high proportions of animal-derived products to more plant-oriented diets can significantly lower environmental impacts. For instance, adopting more sustainable dietary patterns could reduce GHGE and land use by up to 50% (9), or even 70–80%, and water use by as much as 50%, with median reductions of 20–30% across studies (7). A recent large-scale modeling study from the UK underpins this finding, demonstrating that the dietary environmental impacts of vegans are only a quarter compared to high meat-eaters for GHGE and LU, and used less than half of water. Eutrophication and biodiversity loss were also notably lower. All environmental indicators increased with higher consumption of animal-derived foods, independent of production methods and sourcing (10). These environmental benefits were accompanied by improvements in public health outcomes (7, 9). Based on data from the European Prospective Investigation into Cancer and Nutrition (EPIC) cohort, Laine et al. (11) highlighted the inherent benefit of sustainable diets for both health and environmental outcomes. Among 443,991 EPIC-participants, higher dietary GHGE and LU were associated with increased risks of all-cause mortality (adjusted hazard ratio 1.13 [95% CI 1.10–1.16] and 1.18 [95% CI 1.15–1.21] for GHGE and LU, respectively) and cancer incidence (adjusted hazard ratio 1.11 [95% CI 1.09–1.14] and 1.13 [95% CI 1.10–1.15] for GHGE and LU, respectively) (11). In this study, dietary shifts were modeled toward a plant-oriented diet, the Planetary Health Diet, which was introduced by the EAT-Lancet Commission (1). A better alignment with the reference diet led to an estimated prevention of up to 19–63% deaths and 10–39% incidences of cancers over 20 years, while simultaneously reducing dietary GHGE by up to 50% and LU by up to 62% (11).

In Europe, dietary emissions vary widely due to differences in food consumption habits and dietary patterns (12). Men typically emit more GHGE per day than women, largely due to higher energy and meat intake (12–16). Stratified by sex, women emitted 2.9 kg CO_2_eq per day and men 3.6 kg in Sweden (15), while in the Netherlands, emissions were 3.7 kg CO_2_eq for women and 4.8 kg for men (16). Across other European countries, the reported dietary GHGE values were, on average, 6.0 kg CO_2_eq per day in France (17), 4.3 kg CO_2_eq in Ireland (18), 5.2 kg CO_2_eq in Italy (17), and 5.4 kg CO_2_eq in Denmark (17), while Switzerland had a median of 3.25 kg CO_2_eq per day (13). Comparing these figures is challenging due to differences in methodologies. Results are substantially affected by variations in dietary assessment tools (e.g., 24-h recalls or food frequency questionnaires [FFQs]), sampling units (individual, household, or region), measurement units (daily, annual, or energy-adjusted), system boundaries, and the sustainability databases used. Research on dietary emissions in Germany remains limited, with only a few studies providing insights into the environmental impact of food consumption. Koelman et al. (19) reported energy-adjusted emissions of 6.6 kg CO_2_eq per 2000 kcal for men and 7.0 kg CO_2_eq for women in Eastern Germany, with dairy, meat, and beverages as the primary contributors. Notably, energy-adjusted emissions were higher in women. Against this, Meier and Christen used data from the 2nd German Nutritional Food Consumption Survey from 2006 and found that men’s diets had 25% higher emissions and 24% higher LU than women’s per 2000 kcal, primarily driven by greater meat intakes. The WFP was 11% higher in women (20). Treu et al. (21) compared average conventional and organic diets in Germany, observing similar GHGE but 40% higher land use for organic diets, which contained less meat and more plant-based foods. Eberle and Fels (22) modeled German food consumption mainly using national statistics, identifying 2.7 t CO_2_eq per person per year as the environmental burden, with animal products and food losses as key contributors.

These studies highlight the dual benefits of dietary shifts. Nevertheless, the implementation of dietary changes at individual level remains challenging due to the emotional, cultural, and habitual nature of eating (23). Knowledge about the current dietary situation is essential for achieving effective dietary changes. For example, when using a mathematical optimization model to create food-based dietary guidelines, actual intake data are needed to develop a deviation constraint, ensuring that recommended diets remain practical and acceptable to the population. This was demonstrated with the recently published food-based dietary guidelines in Germany (24). Furthermore, there is often a discrepancy between an individual’s awareness of the environmental impact of dietary choices and their actual dietary behaviors (25). This paper describes the status of environment-related sustainability parameters, particularly greenhouse gas emissions (GHGE) but also land use (LU) and water footprint (WFP), using data from the 3rd Bavarian Food Consumption Survey (BVS III). The focus is on comparing lower vs. higher GHGE groups for differences in food choices, and the influence of sociodemographic factors, behaviors, and attitudes toward (environmental) sustainability.

## 2 Methods

### 2.1 Study description

#### 2.1.1 Sampling

The 3rd Bavarian Food Consumption Survey (BVS III) was designed as a statewide representative survey conducted in Bavaria, Germany, between October 2021 and January 2023. The target population comprised all persons aged 18 to 75 years living Bavaria and in private households with sufficient knowledge of the German language. The selection was based on a stratified, multi-stage randomization procedure.

In the first sampling step, a random sample of 60 municipalities was drawn from the entirety of Bavarian municipalities. They were representatively chosen according to the stratification characteristics of administrative district, province (German: *Kreis*), and region type (German: *BIK-Kennzahl*). Each municipality could potentially be sampled multiple times as a sample point (i.e., random sampling with replacement). The BVS III finally comprised 80 sample points of 60 municipalities. A total of 11,000 addresses were requested via the residents’ registration offices. Initially, 100 addresses were obtained per sample point. During the study, the number of addresses was increased to 150 due to low participation rates.

In the second step, 90, respectively 100, addresses per sample point were randomly drawn from the requested addresses. To reach the target sample size of approx. 1,500 participants, a total of 7,449 people were contacted.

#### 2.1.2 Design

The BVS III comprises multiple components. First, the participants were visited at home. The visits consisted of a standardized and interviewer-led computer-assisted personal interview (CAPI), followed by a self-administered computer-assisted self-interview (CASI), Body mass and weight were self-reported. Waist circumference was measured. Dried bloodspots samples (DBS) and blood glucose measures were also taken during the home visits. The CAPI and CASI questionnaires encompassed inquiries regarding various aspects of dietary and health behavior, demographic information, and medical history and included various validated questionnaires (see Supplementary methods). Sustainability was another focal point, with a dedicated section of questions. The selection of questions was based on various publications (26–28) and mainly on Tobler et al. (27).

After home visits, participants completed up to three 24-h dietary recalls via telephone (CATI) over 6 weeks, using GloboDiet^©^, a computer-based survey program derived from EPIC-SOFT^©^ and developed by the International Agency for Research on Cancer (Lyon, France) (29). The Max Rubner-Institute (Karlsruhe, Germany) provided the German version. Food and supplement intakes were recorded on two weekdays and one weekend day. A photo book aided portion size estimations. Data collection was managed by KANTAR (Munich, Germany), and DBS samples were analyzed by Vitas AS (Oslo, Norway). Home visits were conducted from October 2021 to November 2022. Dietary 24-h recalls ended in January 2023. The study design is described in detail in the Supplementary methods and by Rohm et al. (30).

#### 2.1.3 Data quality assurance

To ensure high data quality, attention was devoted to quality assurance procedures. Multiple training sessions and refresher courses were conducted for the interviewers for both home visits and 24-h recalls. A preceding pilot study for the home visits and a pre-test for the 24-h recalls were conducted in August 2020 to facilitate a flawless procedure.

The completeness and plausibility of the collected data from the CAPI and CASI were continuously assessed during the field phase. Due to the computer guidance through and programming of the questionnaire, permissible value ranges and answer options were predefined, and the filter guidance was predetermined, minimizing the possibility of incorrect entries from the start. Minor plausibility corrections were made as needed. The dataset for CAPI and CASI was additionally examined regarding completeness of variables, variable and value labels, value ranges, distribution, and plausibility of values at the beginning, midpoint, and end of the field phase. Minor necessary corrections were made as needed. The nutritional data collection via GloboDiet^©^ enabled a preliminary assessment of completeness and plausibility per participant, facilitated by predefined value ranges for food and recipe portions. GloboDiet^©^ issued alerts during data entry for missing or unusually high inputs and reminders for commonly forgotten items during recall interviews. Before concluding the interview, summary data, including energy and macronutrient intake, were presented, juxtaposed with long-term energy requirements. Warning messages were prompted for intake deviations from these requirements. During the CATI data collection, an interim dataset was reviewed to assess the plausibility of the data. After the final data handover, refined data validation and preparation were conducted, including correcting erroneous inputs and content-based plausibility checks. All corrections were documented.

#### 2.1.4 Data analysis

##### 2.1.4.1 Weighting factors

To ensure representativeness for the Bavarian population, weighting factors for the data from the home visits and dietary assessments were generated based on several criteria. These included administrative district, education level, and combinations of education and age as well as gender and age. Education was classified into three levels: (1) no qualification, lower secondary, or elementary school; (2) secondary education without a high school diploma (“Abitur”) or currently in school; and (3) high school diploma (“Abitur”), university entrance qualification, or university degree. Cases with no information or other forms of education were assigned a factor of 1 (i.e., they were not weighted). The reference was the German 2020 micro census, i.e., an extrapolation of the data for Bavaria (31).

##### 2.1.4.2 Estimates of energy, nutrient, and food intake

Upon completion of the dietary surveys and to assess nutrient intakes, reported foods were matched with the German Nutrient Database (BLS 3.02; German: *Bundeslebensmittelschlüssel 3.02*) (32). Composite dishes and recipes were deconstructed using their individual ingredients based on the recipes from the BLS 3.02. Subsequently, all food items were categorized into food groups on two levels of detail (Table 1).

**Table 1:** Food groups in the BVS III.

Level 1 food group	Level 2 food group	Description
Meat	Meat, uncategorized	Including uncategorized cooked meat, mixed (mince) meat
	Beef	Variety of beef cuts
	Veal	Variety of veal cuts
	Pork	Variety of pork cuts
	Mutton/Lamb	Variety of mutton and lamb cuts
	Horse-, goat-, rabbit meat, and game mammals	Variety of horse, goat, rabbit, or game mammal cuts
	Poultry and game poultry	Variety of poultry or game poultry cuts
	Variety meat and offal	Liver, tongue, heart meat from various animals
Meat and sausage products	Sausage and sausage products	Including cold cuts, raw sausages, cured sausages
	Ham and cured meat	Including mainly pork ham, beef ham, specialty ham
	Canned meat	Aspics
Fish and fish products	Fish, fresh and frozen	Variety of fish and seafood
	Canned fish	Including tinned, canned, preserved, marinated fish and seafood
	Other fish products	Breaded fish and fish products
Eggs	Eggs	Including chicken eggs, egg whites, egg yolks
Milk and dairy products	Milk	Cow’s milk with different fat contents, condensed milk
	Cream	Including heavy cream, crème fraîche
	Cream cheese, quark	Including cream cheese and quark with different fat contents and add-ins, cottage cheese
	Fermented dairy products	Including yoghurt, kefir, soured milk, ayran with and without add-ins
	Other milk-based and dairy products	Butter milk, milk powder, milk-based drinks
	Cheese	Variety of hard, semi-hard, and soft cheeses
Butter	Butter	Butter and clarified butter
Cooking oils and fats (excluding butter)	Margarine	Variety of margarines
	Plant-based fats and oils	Including olive, rape seed, sunflower seed, linseed oil
	Mayonnaise and other fat-based products	Including mayonnaise, remoulade, mayonnaise as salad dressing
	Animal-based fats and oils	Lard from various animals
	Cooking fats and oils, uncategorized	Uncategorized fats and oils
Bread and bakery products	White bread, crispbread, bread rolls	Including white bread, pretzels, flatbread, rolls, baguette
	Other breads	Including brown, gray, spelt bread
	Baked goods and pastries	Including cakes, muffins, tartes, cookies, gingerbread, crackers
Grain-based staple foods	Flour	Including wheat, oat, spelt, barley, millet flour
	Rice	Rice, basmati rice
	Grains (excluding rice)	Including oats, quinoa, millet flakes and kernels
	Other grain products	Including puffed grains, breakfast cereals, popcorn
	Pasta products	Including pasta with and without egg content, filled pasta
Wholegrain products	Wholegrain pasta products	Whole grain pasta
	Muesli	Various muesli mixes
	Wholegrain bread and bread rolls	Wholegrain bread and rolls
Potatoes and potato products	Potatoes, fresh	Potatoes peeled and unpeeled
	Potato products	Including gnocchi, croquettes, hash browns, potato dumplings
Vegetables	Vegetables, uncategorized	Various mixed vegetables
	Salad vegetables	Different salad varieties
	Leafy and stalk vegetables	Includes spinach, fennel, celery
	Cabbage vegetables	Including broccoli, Brussel sprouts, sauerkraut, cauliflower
	Sprout and leek vegetables	Including onion, leek, garlic, bean sprouts
	Fruit vegetables	Including tomato, cucumber, bell pepper, pumpkin, eggplant, zucchini
	Root and tuber vegetables	Including carrot, radish, beetroot
	Oil fruits	Olives
	Mushrooms	Including button, wild, chanterelle mushrooms
	Vegetable products	Including vegetarian pâté, patties
Legumes and pulses	Legumes and pulses	Including chickpeas, various beans, lentils, peas, green beans, snow peas
Fruits	Fruits, uncategorized	Mixed fruits and fruit products
	Pome fruits	Apples, pears, quinces
	Stone fruits	Including apricots, nectarines, plums, cherries, peaches
	Berries	Including mixed berries, grapes, blue berries, raspberries
	Wild fruits	Cranberry products
	Raisins	Raisins
	Tropical fruits	Including bananas, avocados, kiwis, mangos
	Citrus fruits	Including oranges, lemons, clementines
	Canned fruits	Apple sauce and various compotes
Nuts, kernels, and seeds	Nuts, kernels, and seeds	Including almonds, linseed, cashew nuts, peanuts, coconut
Sugars and sweeteners	Sugars	Sugars
	Sweeteners	Sweeteners and sugar substitutes (e.g., xylitol)
Marmalade, jam, and jelly	Marmalade, jam, and jelly	Various marmalades, jams, jellies
Sweets	Cocoa and cocoa drink powders	Cocoa and cocoa drink powders sweetened and unsweetened
	Chocolates and chocolate products	Including chocolate bars, pralines, filled chocolates
	Confectionery and other sweets	Including gummies, cereal bars, drops
	Ice cream	Various ice creams, sorbets, fruit ice creams
	Honey and sweet spreads	Honey, sirups, chocolate spreads
Seasonings and other ingredients	Seasonings and other ingredients	Including spices, condiments, vinegar
Non-alcoholic beverages	Fruit and vegetable juices	Including juices from various fruits
	Table water	Drinking water, table water, mineral water
	Juice spritzers	Including juices mixed with water from various fruits
	Sodas and lemonades	Including cola beverages, lemonades, soft drinks with and without sugar
	Other non-alcoholic beverages	Including alcohol-free beer, alcohol-free wine, iced tea
	Coffee substitutes	Coffee substitute drinks, malt drinks
Alcoholic beverages	Spirits	Various spirits
	Beer	Various beers and mixed beer drinks
	Liqueurs and cocktails	Including fruit, herbal, bitter liqueurs, cocktails
	Wine and sparkling wine	Including red, white, sparkling, mulled wine
Roasted coffee	Roasted coffee	Coffee, instant coffee, espresso
Tea and other infusions	Tea	Black, green, rooibos tea
	Fruit and herbal tea	Fruit and herbal tea with and without sugar and milk
Soups and sauces	Soups and sauces	Including gravy, soup stock, uncategorized soups, other sauces
Substitute products	Milk substitutes	Including soy drink sweetened and unsweetened
	Meat substitutes	Including tofu, soy patties, soy sausage
Desserts and other sweet dishes	Desserts and other sweet dishes	Semolina custards, mousses, sweet puddings

Only participants who completed at least two 24-h dietary recalls were included in the food intake analyses for this paper. Questionnaire-based variables were analyzed using the total study population (*N* = 1,503), with occasionally missing data points.

### 2.2 Measures

Data on sex, age, body height and mass, waist circumference, education level, net household income, and attitudes toward sustainability were used, alongside dietary intake data of the 24-h recalls. Participants self-reported their diet type as vegetarian, vegan, no special diet, or other. Vegans and vegetarians were collectively categorized as plant-based diets, while those with no special diet or other classifications were grouped as omnivorous. For the sociodemographic description of the study population, the variables civil status, living situation, employment, smoking, and physical activity were included, Physical activity groups were determined as described by Gerrior et al. (33).

The equivalized net income was computed using the modified OECD equivalence scale (34). The net household income data were assessed using predefined ranges. The median of the range was used as the metric value for calculation. The maximum value of the monthly net household income was presented in text form as “7,000 Euro and more.” This was interpreted as 7,500 Euro/month. Similarly, the minimum was described as “below 500 Euro” and set to 250 Euro/month.

The dietary data were converted into weekday-weighted averages to account for variations in diet across different days. Individuals with a total energy intake to basal metabolic rate ratio below 0.6 were identified as underreporters and excluded. As there were no cases with a ratio of total energy intake to basal metabolic rate greater than 2.4, overreporters were not excluded. The basal metabolic rate was calculated using World Health Organization (WHO) equations (35). Dietary data were linked to two sustainability metric databases: SHARP-ID (36) for dietary GHGE (in kg carbon dioxide equivalents [CO_2_eq]) and LU (in m^2^*yr), and SuEatableLife (37) for WFP (in kiloliters).

The SHARP-ID database estimates GHGE and LU per kilogram of food as consumed based on dietary data from four European countries (Denmark, Czech Republic, Italy and France). The system boundaries applied within the SHARP-ID encompass the entire life cycle of food products, including primary production, processing, packaging, transport, and consumption (36); and were supplemented with the recorded cooking method by the participants of the BVS III. For animal-derived foods, GHGE and LU were estimated up to the farm gate using the CAPRI model, which provides a cradle-to-gate life-cycle assessment (LCA) of livestock products (meat, milk, and eggs) (5). The EU-average was primarily used as source for animal-derived foods within the SHARP-ID (36). Other sources for primary production impacts for other foods were the Agri-Footprint 2.0 (38, 39) and Ecoinvent 3.3 (40). More detail of the development of the SHARP-ID are comprehensibly described elsewhere by Mertens et al. (36).

The SuEATableLife database provides the WFP for food, based on literature, public reports, and Environmental Product Declarations. The system boundaries applied within the database include the life cycle up to the distribution center (primary production, processing, packaging, and transport), excluding post-market stages, i.e., consumption (37). Hence, the recorded cooking method by BVS III participants was not included in WFP calculations. More details are described by Petersson et al. (37).

Participants were categorized into quintiles based on their GHGE per 2,500 kcal.

### 2.3 Statistical analysis

Descriptive statistics were used for sociodemographic characteristics, with standard deviations (SD) for unweighted and standard errors (SE) for weighted data. Wilcoxon’s rank-sum test was used for two-group comparisons, and Kruskal–Wallis rank-sum test for more than two groups, adjusting for survey samples. Categorical variables were compared using the chi-squared test with Rao and Scott’s correction for weighted data or Pearson’s chi-squared and Fisher’s exact tests for unweighted data. Generalized linear models were used for linear trend analyses with ordered factors. Food group intakes, GHGE, LU, and WFP were adjusted to 2,500 kcal. Weighted linear regression was performed to assess the association of sociodemographic factors and diet type with dietary GHGE, LU, and WFP. Heteroscedasticity-consistent standard errors type 3 were used when necessary. Outliers, defined as residuals exceeding two standard deviations, were removed to maintain normality in the models. Marginal means were calculated from regression models. The estimate for the marginal mean represents the predicted average for each group while averaging over the other independent variables. This approach accounts for confounding factors for adjusted comparisons between groups. Data were weighted to reflect the Bavarian population. All statistical analyses and graphical depictions were performed using the statistical software R version 4.4.0 (41).

## 3 Results

### 3.1 Study characteristics

#### 3.1.1 Response

**Figure 1:**
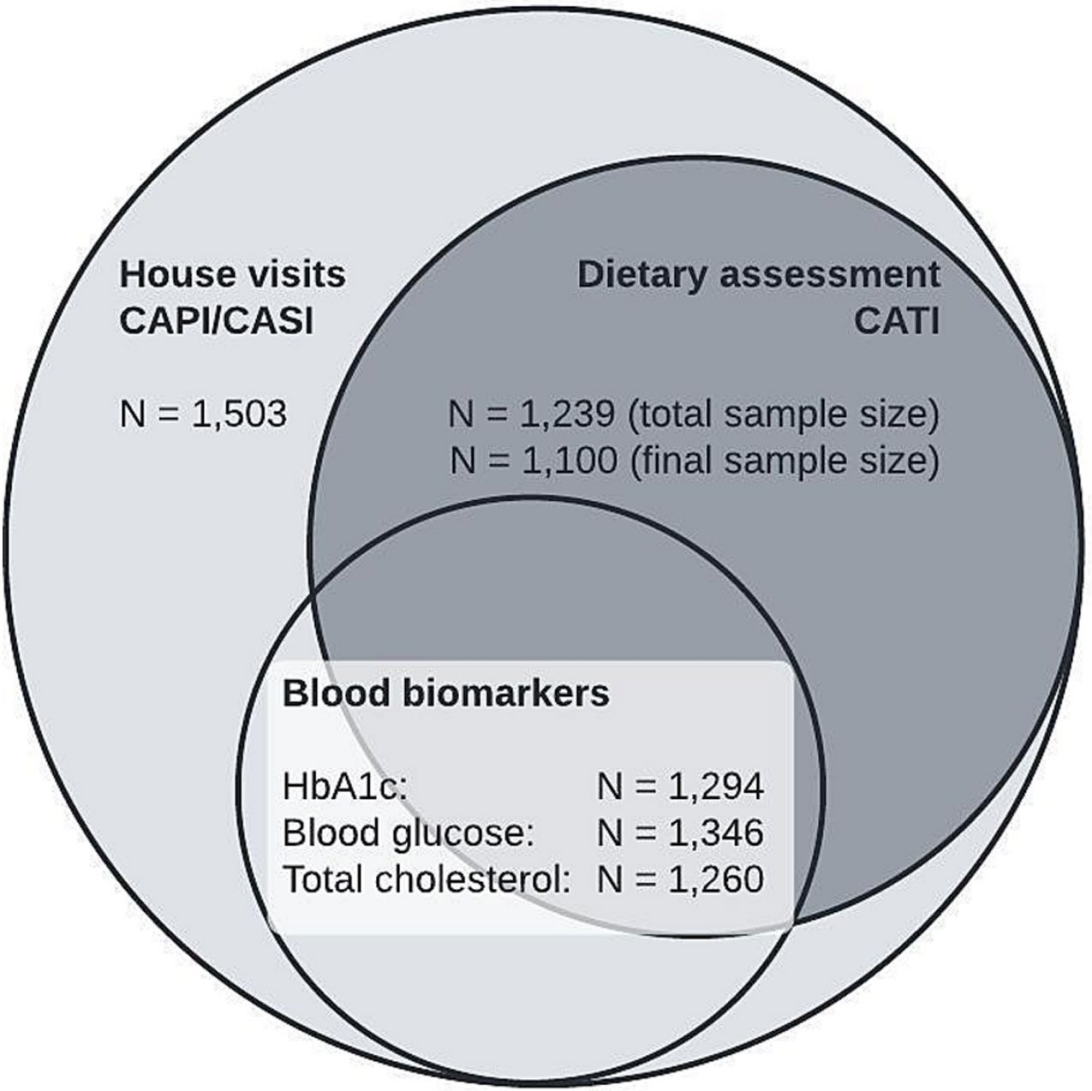
Study population by survey components of the BVS III.

Out of the 7,449 individuals contacted, the cleaned gross sample, excluding quality-neutral dropouts (QND), consisted of 5,770 individuals. From this, 1,503 (26.0% response rate) participated in the CAPI/CASI during home visits. During these visits, waist circumference was measured for 1,448 participants, blood glucose levels for 1,346; HbA1c for 1,294; and total cholesterol for 1,260. Of the 1,503 participants, 1,239 completed at least one 24-h recall, and 1,148 (76.4%) at least two 24-h recalls. Only the latter were considered for the estimation of dietary intake. Of these, 48 people were excluded as underreporters, resulting in a final sample size for the dietary assessment of 1,100 participants (Figure 1). 17.6% of the participants who completed the house visit were not reached for the dietary assessment. Some variables had missing data points. The sample sizes for each variable were as follows: waist circumference (*N* = 1,448), education (*N* = 1,502), equivalized net household income (*N* = 1,380), civil status (*N* = 1,501), employment (*N* = 1,502), and smoking status (*N* = 1,502). Absolute numbers may differ when weighting is applied. Further details are shown in the Supplementary results.

#### 3.1.2 Demographics

The total BVS III study sample is described in Table 2 without having applied weighting factors. The sample consisted of 1,503 participants, with 46% male (*n* = 688) and 54% female (*n* = 815). The mean age of the participants was 48.0 years, with no significant difference between males and females (*p* = 0.484). Age distribution across the defined age groups (18–24, 25–34, 35–50, 51–64, ≥65 years) was similar between sexes (*p* = 0.920).

**Table 2:** Sociodemographic characteristics of the full study population (*N* = 1,503).

Variable	*N*	Overall, *N* = 1,503 (100%)^1^	Sex		*p*-value^2^
			Male, *N* = 688 (46%)^1^	Female, *N* = 815 (54%)^1^	
Age (in years)	1,503	48.0 ± 15.1	47.7 ± 15.3	48.3 ± 14.9	0.484
Age group (in years)	1,503				>0.9
18–24		109 (7%)	50 (7%)	59 (7%)	
25–34		239 (16%)	115 (17%)	124 (15%)	
35–50		436 (29%)	193 (28%)	243 (30%)	
51–64		479 (32%)	220 (32%)	259 (32%)	
≥65		240 (16%)	110 (16%)	130 (16%)	
BMI (in kg/m^2^)	1,503	25.9 ± 5.0	26.8 ± 4.5	25.2 ± 5.3	<0.001
BMI group^3^	1,503				<0.001
Underweight		29 (2%)	4 (1%)	25 (3%)	
Normal weight		708 (47%)	265 (39%)	443 (54%)	
Pre-obesity		497 (33%)	275 (40%)	222 (27%)	
Obesity		269 (18%)	144 (21%)	125 (15%)	
Waist circumference (in cm)	1,448	93.3 ± 15.3	99.8 ± 13.6	87.8 ± 14.4	<0.001
Education	1,502				0.004
Low		339 (23%)	162 (24%)	177 (22%)	
Middle		435 (29%)	170 (25%)	265 (33%)	
High		728 (48%)	355 (52%)	373 (46%)	
Equivalized net household income (in Euro/month)	1,380	2,051.8 ± 1,488.0	2,054.6 ± 1,467.7	2,049.5 ± 1,505.6	0.703
Civil status	1,501				<0.001
Single		221 (15%)	114 (17%)	107 (13%)	
Unmarried – in a partnership		212 (14%)	103 (15%)	109 (13%)	
Married		922 (61%)	427 (62%)	495 (61%)	
Widowed		38 (3%)	5 (1%)	33 (4%)	
Divorced		108 (7%)	38 (6%)	70 (9%)	
Living situation	1,503				0.394
Living alone in a private household		240 (16%)	108 (16%)	132 (16%)	
Living in a private household with family/friends or other persons		1,247 (83%)	570 (83%)	677 (83%)	
Other		16 (1%)	10 (1%)	6 (1%)	
Employment	1,502				<0.001
Employed		988 (66%)	475 (69%)	513 (63%)	
Marginally, occasionally or irregularly employed		42 (3%)	8 (1%)	34 (4%)	
In vocational training/apprenticeship/retraining		22 (1%)	14 (2%)	8 (1%)	
Currently not employed: unemployed or job-seeking, on parental leave		84 (6%)	28 (4%)	56 (7%)	
Retired, pensioner, homemaker		310 (21%)	136 (20%)	174 (21%)	
Other (e.g., pupil, student, assisting family member)		56 (4%)	26 (4%)	30 (4%)	
Smoking	1,502				0.002
Never		764 (51%)	320 (47%)	444 (54%)	
Currently		289 (19%)	155 (23%)	134 (16%)	
In the past		449 (30%)	212 (31%)	237 (29%)	
Physical activity group^4^	1,503				<0.001
Sedentary		415 (28%)	157 (23%)	258 (32%)	
Low active		379 (25%)	155 (23%)	224 (27%)	
Active		331 (22%)	152 (22%)	179 (22%)	
Very active		378 (25%)	224 (33%)	154 (19%)	
Diet type	1,503				<0.001
Omnivorous		1,385 (92%)	653 (95%)	732 (90%)	
Vegetarian or vegan		118 (8%)	35 (5%)	83 (10%)	

^1^Mean ± SD; *n* (%).

^2Wilcoxon rank sum test; Kruskal–Wallis rank sum test; Pearson’s Chi-squared test.^

^3^According to the definition of the WHO (79).

^4^According to Gerrior et al. (33).

Most participants had a higher education (48%). Education levels differed significantly by sex (*p* = 0.004). A higher proportion of males (52%) had a higher education than females (46%). Conversely, a greater proportion of females had midlevel education.

Married individuals made up 61% of the sample. A larger proportion of males were single (17% vs. 13% of females), while more females were divorced (9% vs. 6% of males) or widowed (4% vs. 1% of males) (*p* < 0.001).

Most participants lived in private households with family or friends (83%), with a small proportion living alone (16%) or with other arrangements (1%).

Two-thirds of the subjects were employed. Employment status showed significant differences between sexes (*p* < 0.001). A higher percentage of males were employed (69% vs. 63% in females), while more females were marginally, occasionally, or irregularly employed (4% vs. 1% in males). Additionally, more females were currently unemployed, including those on parental leave (7% vs. 4% in males).

The demographic description of the study sample that completed the dietary assessment with at least two 24-h recalls is shown in Supplementary Table 1 and was comparable to the complete study sample with only minor deviations. Weighting factors were applied to ensure further results were representative of the Bavarian population. Consequently, further analyses used weighted data to make inferences about the Bavarian population.

The average BMI in the Bavarian population was 25.9 kg/m^2^ (Table 3). Half of the population was overweight, i.e., they had a BMI ≥25 kg/m^2^. The proportion of overweight people was lower in females (41%) than in males (59%, *p* < 0.001). The average BMI in females was 25.4 kg/m^2^ and significantly lower than the males’ average of 26.5 kg/m^2^ (*p* < 0.001). Nearly a quarter of the population were smokers during the survey. Around 50% of the individuals were physically active or very active (Table 3 and Supplementary Table 2).

**Table 3:** Demographic characteristics in the BVS III (*N* = 1,503).

Variable	*N*	Overall, *N* = 1,503 (100%)^1^	Sex		*p*-value^2^
			Male, N = 756 (50%)^1^	Female, *N* = 747 (50%)^1^	
Age (in years)	1,503	46.3 ± 0.6	45.7 ± 0.9	46.8 ± 0.8	0.366
Age group (in years)	1,503				>0.9
18–24		157 (10%)	82 (11%)	74 (10%)	
25–34		268 (18%)	140 (19%)	128 (17%)	
35–50		436 (29%)	215 (28%)	221 (30%)	
51–64		421 (28%)	215 (28%)	206 (28%)	
≥65		221 (15%)	104 (14%)	117 (16%)	
BMI (in kg/m^2^)	1,503	25.9 ± 0.2	26.5 ± 0.3	25.4 ± 0.3	<0.001
BMI group^3^	1,503				<0.001
Underweight		23 (2%)	2 (0%)	21 (3%)	
Normal weight		722 (48%)	306 (40%)	417 (56%)	
Pre-obesity		480 (32%)	297 (39%)	182 (24%)	
Obesity		278 (18%)	151 (20%)	127 (17%)	
Waist circumference (in cm)	1,434	93.6 ± 0.6	99.2 ± 0.9	87.8 ± 0.8	<0.001
Education	1,498				0.241
Low		530 (35%)	288 (38%)	242 (32%)	
Middle		429 (29%)	199 (27%)	229 (31%)	
High		540 (36%)	264 (35%)	276 (37%)	
Equivalized net household income (in Euro/month)	1,353	2,109.2 ± 63.9	2,092.5 ± 96.5	2,125.5 ± 84.2	0.818
Civil status	1,501				0.002
Single		260 (17%)	163 (22%)	97 (13%)	
Unmarried – in a partnership		221 (15%)	99 (13%)	122 (16%)	
Married		885 (59%)	445 (59%)	440 (59%)	
Widowed		39 (3%)	7 (1%)	32 (4%)	
Divorced		96 (6%)	41 (5%)	56 (7%)	
Living situation	1,503				0.218
Living alone in a private household		258 (17%)	134 (18%)	123 (16%)	
Living in a private household with family/friends or other persons		1,218 (81%)	601 (79%)	617 (83%)	
Other		28 (2%)	21 (3%)	7 (1%)	
Employment	1,498				0.198
Employed		972 (65%)	510 (68%)	463 (62%)	
Marginally, occasionally or irregularly employed		39 (3%)	13 (2%)	26 (4%)	
In vocational training/apprenticeship/retraining		31 (2%)	20 (3%)	11 (1%)	
Currently not employed: unemployed or job-seeking, on parental leave		92 (6%)	39 (5%)	53 (7%)	
Retired, pensioner, homemaker		304 (20%)	134 (18%)	170 (23%)	
Other (e.g., pupil, student, assisting family member)		59 (4%)	36 (5%)	24 (3%)	
Smoking	1,502				0.199
Never		694 (46%)	331 (44%)	363 (49%)	
Currently		362 (24%)	204 (27%)	158 (21%)	
In the past		446 (30%)	221 (29%)	226 (30%)	
Physical activity group^4^	1,503				<0.001
Sedentary		381 (25%)	156 (21%)	225 (30%)	
Low active		337 (22%)	137 (18%)	200 (27%)	
Active		323 (21%)	154 (20%)	169 (23%)	
Very active		462 (31%)	309 (41%)	153 (20%)	
Diet type	1,503				0.016
Omnivorous		1,408 (94%)	724 (96%)	685 (92%)	
Vegetarian or vegan		95 (6%)	33 (4%)	62 (8%)	

Data are weighted to represent the Bavarian population.

^1^Mean ± SE; *n* (%).

^2Design-based Kruskal–Wallis rank sum test; design-based Wilcoxon rank sum test; chi-squared test with Rao and Scott’s second-order correction.^

^3^According to the definition of the WHO (79).

^4^According to Gerrior et al. (33).

### 3.2 Dietary sustainability

#### 3.2.1 Dietary greenhouse gas emissions, land, and water use in Bavaria

Table 4 presents data on GHGE, LU, and the WFP differentiated by various demographic and dietary groups.

**Table 4:** Diet-related environmental sustainability metrics among various demographic and dietary groups in Bavaria.

Characteristic	GHGE in kg CO_2_eq^1^		LU in m^2^*yr^1^		WFP in kilolitres^1^	
	Per day	Per 2,500 kcal	Per day	Per 2,500 kcal	Per day	Per 2,500 kcal
Overall	4.42 ± 0.07	6.14 ± 0.08	5.46 ± 0.11	7.50 ± 0.12	3.12 ± 0.05	4.39 ± 0.06
Sex						
Male	4.89 ± 0.10	6.17 ± 0.12	6.28 ± 0.15	7.91 ± 0.18	3.27 ± 0.07	4.13 ± 0.07
Female	3.94 ± 0.09	6.11 ± 0.10	4.61 ± 0.12	7.08 ± 0.14	2.97 ± 0.07	4.67 ± 0.09
*p*-value^2^	<0.001	0.656	<0.001	<0.001	0.002	<0.001
Age group (in years)						
18–24	4.28 ± 0.33	6.30 ± 0.45	5.60 ± 0.54	8.06 ± 0.74	3.12 ± 0.27	4.64 ± 0.29
25–34	4.32 ± 0.16	5.81 ± 0.17	5.28 ± 0.22	7.05 ± 0.25	2.99 ± 0.11	4.04 ± 0.12
35–50	4.34 ± 0.13	6.06 ± 0.14	5.35 ± 0.18	7.42 ± 0.20	3.17 ± 0.09	4.50 ± 0.11
51–64	4.53 ± 0.13	6.17 ± 0.12	5.59 ± 0.19	7.52 ± 0.19	3.16 ± 0.08	4.37 ± 0.09
≥65	4.57 ± 0.16	6.55 ± 0.14	5.54 ± 0.23	7.83 ± 0.20	3.11 ± 0.09	4.53 ± 0.13
*p*-value^3^	0.697	0.006	0.864	0.047	0.441	0.036
*p*-trend^4^	0.301	0.369	0.875	0.969	0.789	0.823
Education						
Lower	4.64 ± 0.13	6.42 ± 0.16	5.81 ± 0.19	7.99 ± 0.25	3.15 ± 0.09	4.37 ± 0.11
Medium	4.30 ± 0.14	6.06 ± 0.13	5.32 ± 0.22	7.40 ± 0.20	3.13 ± 0.11	4.47 ± 0.11
Higher	4.32 ± 0.10	5.96 ± 0.10	5.25 ± 0.14	7.15 ± 0.16	3.10 ± 0.06	4.36 ± 0.08
*p*-value^3^	0.055	0.031	0.023	0.002	0.924	0.555
*p*-trend^4^	0.044	0.015	0.019	0.004	0.646	0.903
BMI group^5^						
Underweight	3.73 ± 0.47	5.86 ± 0.55	4.22 ± 0.64	6.49 ± 0.68	2.69 ± 0.18	4.44 ± 0.21
Normal weight	4.15 ± 0.10	5.85 ± 0.12	4.93 ± 0.14	6.85 ± 0.18	3.06 ± 0.07	4.37 ± 0.09
Pre-obesity	4.63 ± 0.12	6.36 ± 0.13	5.84 ± 0.17	7.97 ± 0.19	3.16 ± 0.09	4.37 ± 0.11
Obesity	4.78 ± 0.18	6.51 ± 0.16	6.22 ± 0.27	8.39 ± 0.24	3.25 ± 0.11	4.49 ± 0.12
*p*-value*^3^*	0.005	<0.001	<0.001	<0.001	0.175	0.281
*p*-trend^4^	0.031	0.169	0.005	0.005	0.010	0.834
Diet type						
Omnivorous	4.50 ± 0.07	6.24 ± 0.08	5.59 ± 0.11	7.67 ± 0.12	3.14 ± 0.05	4.40 ± 0.06
Vegetarian or vegan	3.23 ± 0.17	4.72 ± 0.17	3.55 ± 0.21	5.07 ± 0.14	2.87 ± 0.15	4.23 ± 0.17
*p*-value^2^	<0.001	<0.001	<0.001	<0.001	0.076	0.286

Data are weighted to represent the Bavarian population.

CO_2_eq, carbon dioxide equivalents; GHGE, greenhouse gas emissions; LU, land use; WFP, water footprint.

^1Mean ± SE.^

^2Design-based Wilcoxon rank-sum test.^

^3Design-based Kruskal–Wallis rank-sum test.^

^4Design-based generalized linear model with ordered factor levels.^

^5^According to the definition of the WHO (79).

In Bavaria, the average environmental impact per 2,500 kcal of dietary intake was 6.14 kg CO₂eq for GHGE, 7.50 m^2^*yr. for LU, and 4.39 kiloliters for the WFP. There were notable differences in environmental impacts between the sexes. While GHGE per 2,500 kcal was similar between males (6.17 kg CO₂eq) and females (6.11 kg CO₂eq), LU was significantly higher (*p* < 0.001) in males (7.91 m^2^*yr. per 2,500 kcal) compared to females (7.08 m^2^*yr. per 2,500 kcal). However, the WFP was higher in females, with 4.67 kiloliters per 2,500 kcal compared to 4.13 kiloliters for males.

Plant-based diets resulted in significantly lower GHGE (4.72 kg CO_2_eq per 2,500 kcal) and LU (5.07 m^2^*yr. per 2,500 kcal) compared to omnivore diets (GHGE: 6.24 kg CO_2_eq per 2,500 kcal; LU: 7.67 m^2^*yr. per 2,500 kcal) (*p* < 0.001). The WFP was marginally lower in people following such plant-based diets (4.23 kiloliters per 2,500 kcal) compared to their omnivorous counterparts (4.40 kiloliters per 2,500 kcal), albeit not significantly (*p* = 0.286).

Mean energy intake differed between BMI groups with 1,575 kcal for underweight, 1,797 kcal for normal weight, 1,857 kcal for pre-obese, and 1,854 kcal for obese participant. Unadjusted, all environmental metrics showed higher values in the higher BMI groups, with significant *p*-trends (all *p*-trend ≤0.031, see Table 4). Adjusted to 2,500 kcal, obese individuals had the highest GHGE per 2,500 kcal (6.51 kg CO_2_eq) compared to other BMI groups, with the difference being significant (*p* < 0.001). However, there was no statistically substantiated positive trend (*p*-trend = 0.169) across the BMI groups. LU was also significantly higher (*p* < 0.001) in obese (8.39 m^2^*yr. per 2,500 kcal), and a positive trend was observed (*p*-trend = 0.005). However, no differences were observed across the groups with respect to the WFP.

Differences by education level showed a decreasing trend, with higher education associated with lower GHGE (5.96 kg CO_2_eq; *p*-trend = 0.015) and LU (7.15 m^2^*yr.; *p*-trend = 0.004).

To further analyze the contributing factors to dietary emissions, regression analyses were performed to control for potential confounders. Table 5 displays the result of three weighted linear regression analyses examining the association of sociodemographic factors and diet type with dietary GHGE, LU, and the WFP, all adjusted to 2,500 kcal. The results refined the results of the previous trend analyses in Table 4. For group comparisons, marginal means were used to adjust for other independent variables in the model. The regression models indicated that females had higher GHGE and WFP compared to males when controlling for the other independent variables. The marginal means for GHGE was 5.53 kg CO_2_eq for females and 5.33 kg CO_2_eq for males, with a significant difference (*β* = 0.204, *p* = 0.023). Similarly, the marginal mean for the WFP was 4.33 kiloliters for females and 3.87 kiloliters for males with a significant difference (*β* = 0.466, *p* < 0.001). However, there was no significant difference in LU.

**Table 5:** Weighted linear regression of dietary GHGE, LU, and WFP on sociodemographic factors and diet type.

	GHGE (in kg CO_2_eq)			LU (in m^2^*yr)^1^			WFP (in kiloliters)		
Characteristic	Beta^2^	95% CI^2^	*p*-value^2^	Beta^3^	95% CI^3^	*p*-value^3^	Beta^4^	95% CI^4^	*p*-value^4^
(Intercept)	5.19	4.51, 5.88	<0.001	4.81	3.00, 6.62	<0.001	3.91	3.42, 4.39	<0.001
Sex									
Male	—	—		—	—		—	—	
Female	0.204	0.028, 0.381	0.023	−0.181	−0.591, 0.230	0.388	0.466	0.342, 0.589	<0.001
Age group (in years)									
18–24	—	—		—	—		—	—	
25–34	−0.310	−0.653, 0.033	0.076	−0.387	−1.43, 0.656	0.466	−0.352	−0.599, −0.104	0.005
35–50	−0.402	−0.736, −0.068	0.018	−0.521	−1.56, 0.517	0.325	−0.119	−0.361, 0.123	0.334
51–64	−0.297	−0.640, 0.045	0.089	−0.565	−1.63, 0.501	0.299	−0.167	−0.414, 0.080	0.184
≥65	−0.089	−0.476, 0.297	0.650	−0.171	−1.28, 0.942	0.763	−0.105	−0.380, 0.170	0.453
Waist circumference (in cm)	0.011	0.005, 0.017	<0.001	0.035	0.018, 0.052	<0.001	0.002	−0.002, 0.007	0.307
Education									
Low	—	—		—	—		—	—	
Middle	−0.028	−0.245, 0.190	0.804	−0.381	−0.851, 0.088	0.111	0.068	−0.084, 0.220	0.383
High	−0.033	−0.247, 0.180	0.759	−0.239	−0.708, 0.230	0.317	0.119	−0.030, 0.268	0.118
Equivalized net household income (in 1000 Euro/month)	0.011	−0.045,0.066	0.704	−0.019	−0.171, 0.133	0.808	0.003	−0.035, 0.042	0.861
Diet type									
Omnivorous	—	—		—	—		—	—	
Vegetarian or vegan	−1.38	−1.69, −1.06	<0.001	−1.83	−2.22, −1.45	<0.001	−0.335	−0.560, −0.111	0.003

The table depicts three linear regression analyses. The dependent variables are energy-adjusted to 2,500 kcal. For LU, standard errors of coefficients were estimated using heteroscedasticity-consistent 3 standard errors to account for potential heteroscedasticity in the data. Outliers were removed to allow for normality of residuals. Data are weighted to represent the Bavarian population.

CO_2_eq, carbon dioxide equivalents; GHGE, greenhouse gas emissions; LU, land use; WFP, water footprint.

^1Standard errors of coefficients were estimated using heteroscedasticity-consistent 3 standard errors.^

^2^*R*^2^ = 0.11, adj. *R*^2^ = 0.10, *F*(10, 947) = 11.58, *p* < 0.001.

^3^*R*^2^ = 0.20, adj. *R^2^* = 0.19, *F*(10, 953) = 23.29, *p* < 0.001.

^4^*R*^2^ = 0.07, adj. *R^2^* = 0.08, *F*(10, 945) = 8.10, *p* < 0.001.

Waist circumference was positively associated with GHGE (*β* = 0.011, *p* < 0.001) and LU (*β* = 0.035, *p* < 0.001). Plant-based diets were linked to significantly and considerably lower environmental impact. The marginal means for GHGE for participants adhering to a plant-based diet was 4.74 kg CO_2_eq, compared to omnivores with 6.12 kg CO_2_eq (*β* = −1.38, *p* < 0.001). Likewise, LU was 5.56 m^2^*yr. for plant-based diets compared to omnivores with 7.39 m^2^*yr. (*β* = −1.83, *p* < 0.001), and WFP was 3.93 kiloliters against 4.27 kiloliters (*β* = −0.335, *p* = 0.003). Education and the equalized net income did not have a statistically substantiated effect on any of the outcomes.

#### 3.2.2 Characterization of the GHGE quintiles

Emphasis was placed on GHGE. Five GHGE groups were built by stratifying the participants based on quintiles of their CO_2_eq emissions per 2,500 kcal: Lowest, low, medium, high, and highest. The quintiles are characterized by their environmental sustainability metrics in Table 6 and by their sociodemographic characteristics in Supplementary Table 3. Expectedly, GHGE showed a positive trend across the emission groups, as they were constructed based on the GHGE per 2,500 kcal, from 4.24 to 8.52 kg CO_2_eq per 2,500 kcal, but also LU from 5.31 to 10.52 m^2^*yr. per 2,500 kcal and WU from 3.62 to 5.44 kiloliters per 2,500 kcal (all *p*-trend <0.001).

**Table 6:** Characterization of the GHGE quintiles by GHGE, LU, and WFP.

Characteristic	Overall, *N* = 1,100 (100%)^1^	Quintiles GHGE					*p*-trend^2^
		Lowest, *N* = 220 (20%)^1^	Low *N* = 221 (20%)^1^	Medium *N* = 219 (20%)^1^	High *N* = 221 (20%)^1^	Highest *N* = 219 (20%)^1^	
Dietary GHGE in kg CO_2_eq							
Per day	4.42 ± 0.07	3.17 ± 0.11	4.09 ± 0.13	4.40 ± 0.15	4.98 ± 0.14	5.47 ± 0.14	<0.001
Per 2,500 kcal/d	6.14 ± 0.08	4.24 ± 0.05	5.23 ± 0.02	5.97 ± 0.02	6.76 ± 0.04	8.52 ± 0.15	<0.001
Dietary LU in m^2^*yr							
Per day	5.46 ± 0.11	4.08 ± 0.14	5.10 ± 0.22	5.42 ± 0.26	5.97 ± 0.22	6.73 ± 0.23	<0.001
Per 2,500 kcal/d	7.50 ± 0.12	5.31 ± 0.11	6.40 ± 0.11	7.23 ± 0.16	8.08 ± 0.13	10.52 ± 0.3	<0.001
Dietary WFP in kiloliters							
Per day	3.12 ± 0.05	2.65 ± 0.08	3.04 ± 0.10	3.08 ± 0.09	3.42 ± 0.14	3.42 ± 0.10	<0.001
Per 2,500 kcal/d	4.39 ± 0.06	3.62 ± 0.07	3.94 ± 0.07	4.31 ± 0.14	4.67 ± 0.11	5.44 ± 0.15	<0.001

Data are weighted to represent the Bavarian population.

CO_2_eq, carbon dioxide equivalents; GHGE, greenhouse gas emissions; LU, land use; WFP, water footprint.

^1Mean ± SE.^

^2Design-based generalized linear model with ordered factor levels.^

**Figure 2:**
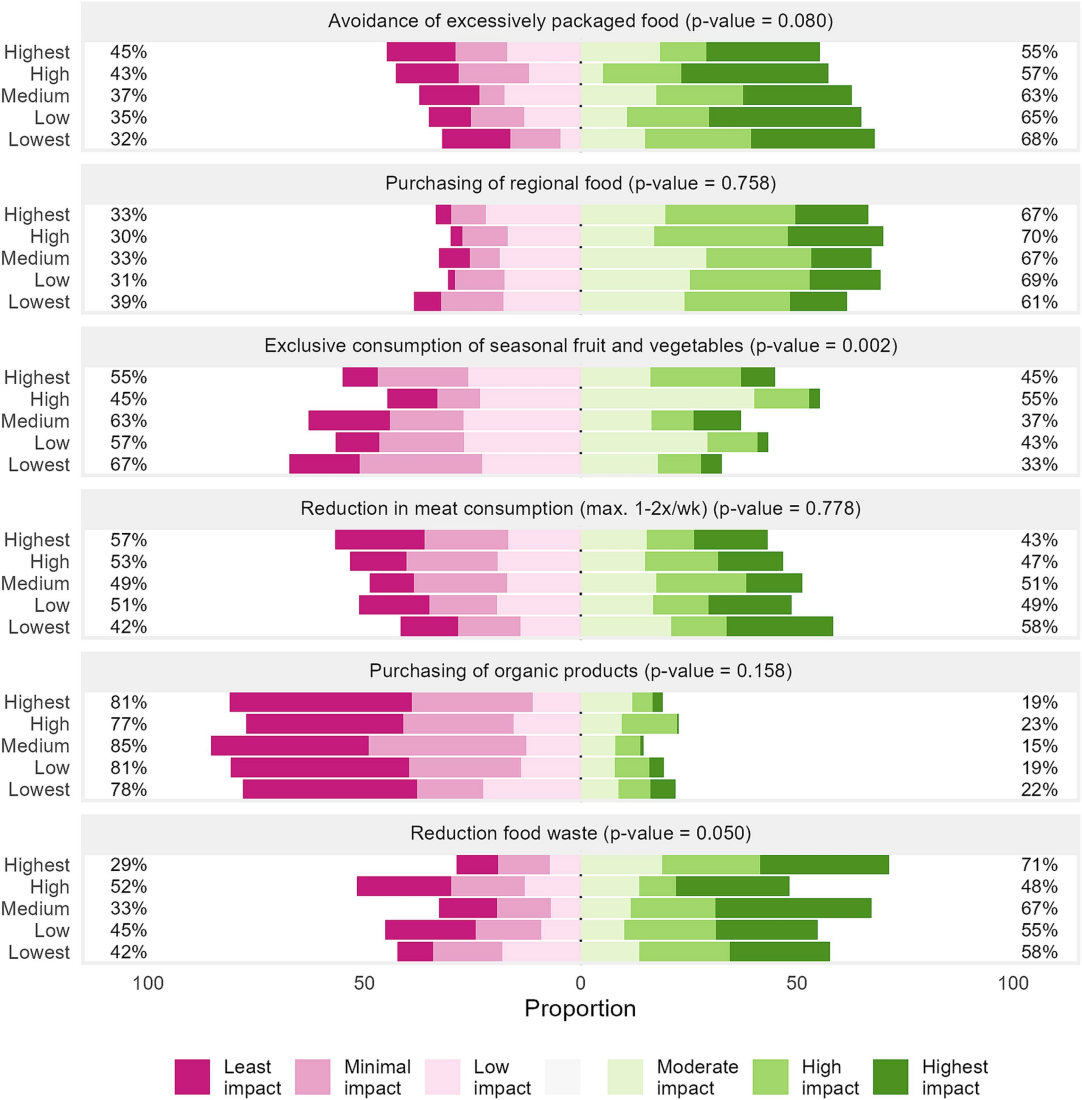
Perceived environmental impact of selected nutritional behaviors (*N* = 1,098) across the GHGE quintiles. Data are weighted to represent the Bavarian population. *p*-values were computed based on chi-squared tests with Rao and Scott’s second-order correction. CO2eq carbon dioxide equivalents, GHGE greenhouse gas emissions.

**Figure 3:**
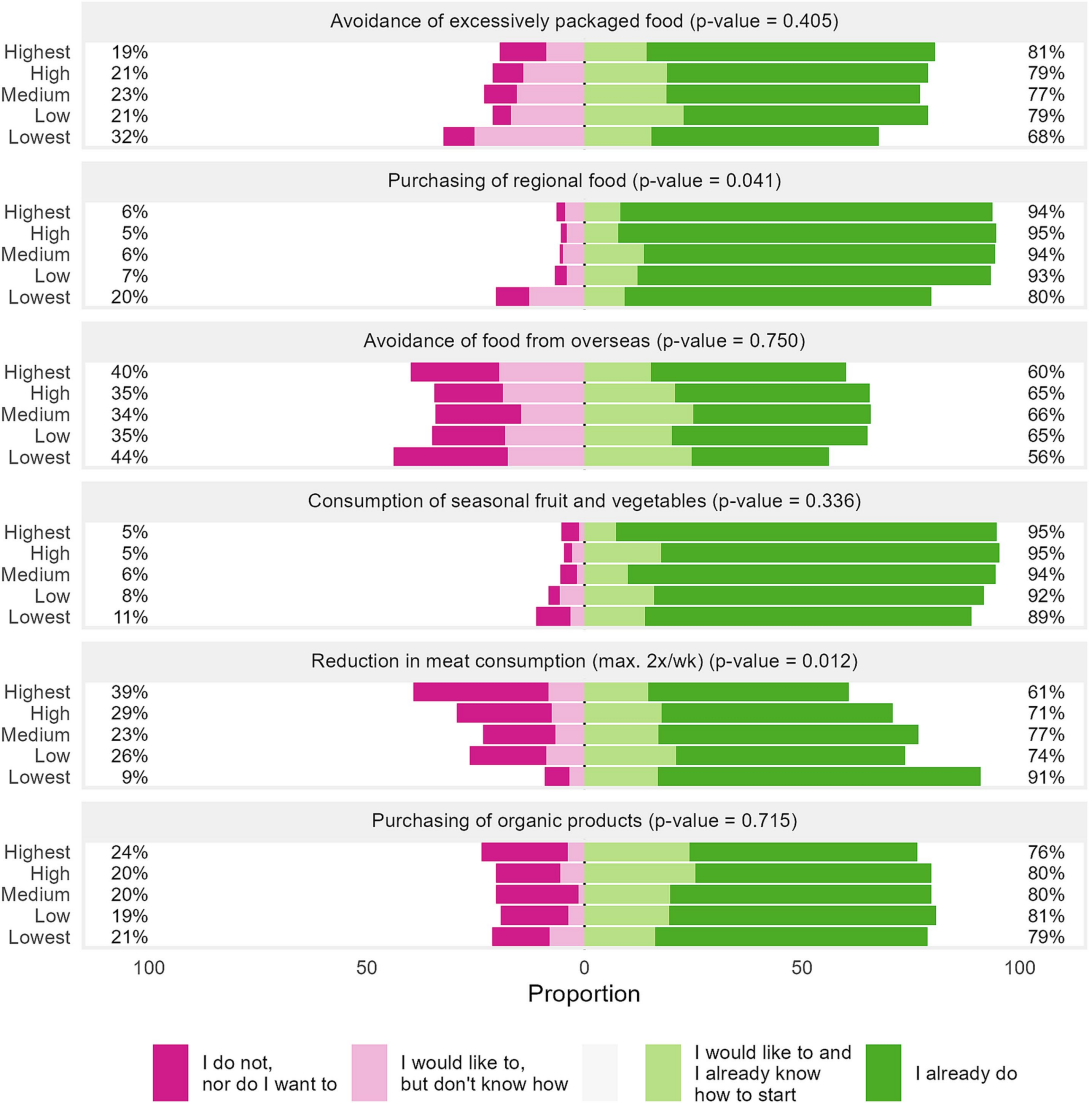
Self-reported extent of selected nutritional behaviors with environmental relevance (*N* = 1,100) across the GHGE quintiles. Data are weighted to represent the Bavarian population. *p*-values were computed based on chi-squared tests with Rao and Scott’s second-order correction. CO_2_eq, carbon dioxide equivalents; GHGE, greenhouse gas emissions.

Figure 2 shows the perceived environmental impact of various nutritional behaviors across the GHGE quintiles. Figure 3 complements these findings by depicting the self-reported implementation of these behaviors. The lowest GHGE quintile perceived the environmental impact of exclusive consumption of seasonal fruits and vegetables as less impactful (33% vs. 37–55%, *p* = 0.002, Figure 2) and were less likely to intend or actually practice purchasing regional food (80% vs. 93–95%, *p* = 0.041, Figure 3). However, they were more inclined to reduce their meat consumption, with 91% either practicing or having the concrete intention to show this behavior, compared to 61–77% among higher emitters (*p* = 0.012, Figure 3). In contrast, the highest emitters placed greater importance on reducing food waste (71% vs. 48–67%, *p* = 0.050, Figure 2) and predominately purchased or intended to buy regional foods (94%, *p* = 0.041, Figure 3).

#### 3.2.3 Contribution of food groups to GHGE and food consumption patterns by GHGE quintiles

**Figure 4:**
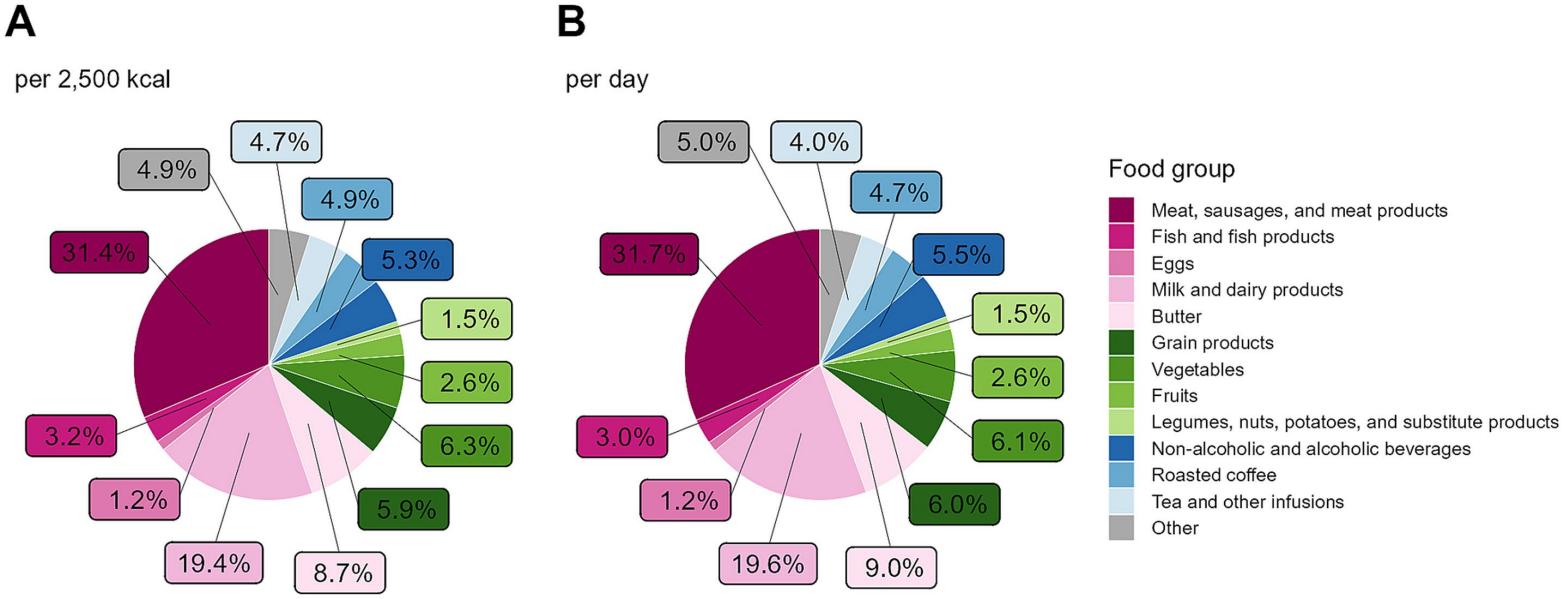
Contribution of food groups to GHGE (*N* = 1,100), presented per 2,500 kcal **(A)** and per day **(B)**. Data are weighted to represent the Bavarian population. GHGE, greenhouse gas emissions.

**Figure 5:**
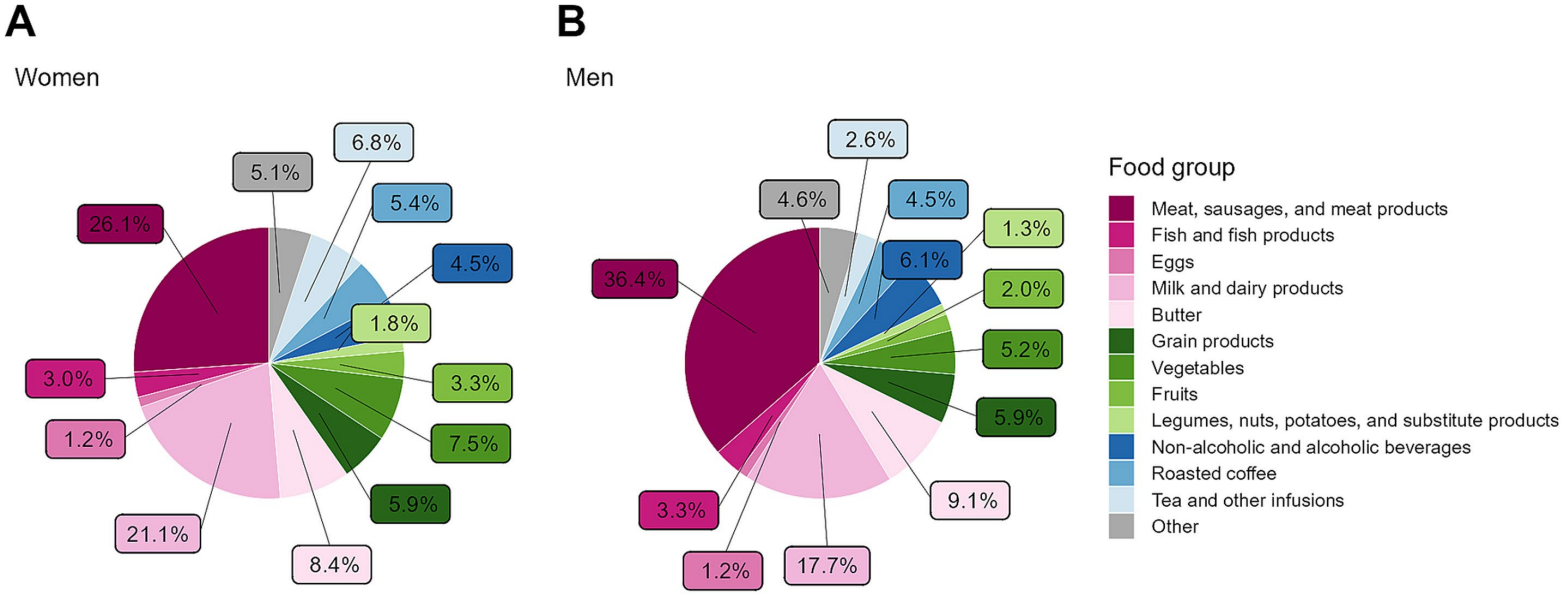
Contribution of food groups to GHGE stratified by sex and adjusted to 2,500 kcal for **(A)** women (*N* = 747) and **(B)** men (*N* = 756). Data are weighted to represent the Bavarian population. GHGE, greenhouse gas emissions.

Figure 4 displays the contribution of different food groups to GHGE. In Figure 4A, the proportional GHGE were standardized to 2,500 kcal to reflect the relative dietary composition, while Figure 4B illustrates GHGE per day without energy adjustment. The overall distribution of GHGE across food groups did not differ greatly between the two approaches and was also similar when stratified by sex (Supplementary Figures 1, 2) or by BMI group (Supplementary Tables 4, 5). Animal-derived foods, i.e., meat, sausages, and meat products; fish and fish products; eggs; milk and dairy products; and butter, accounted for almost two-thirds of dietary GHGE. Among these, meat, sausages, and meat products contribute about 50% to the emissions, regardless of energy-adjustment. Stratified by sex and adjusted to 2,500 kcal (Figure 5), the overall GHGE distribution by sex was generally similar compared to the full study sample. Between the sexes, some differences were, however, notable. Meat, sausages, and meat products contributed to a lower degree to emissions in females (26.1%) compared to males (36.4%), whereas milk and dairy products accounted for a higher proportion in females (21.1%; males: 17.7%). Additionally, the contribution of tea and other infusions, vegetables, fruits, and, to a lesser extent, coffee was higher in females, while the proportion of non-alcoholic and alcoholic beverages (excluding coffee and tea) to GHGE was greater in males. Stratified by BMI groups and adjusted to 2,500 kcal, the consumption of meat, sausage and meat products was strikingly higher in pre-obese (36.0%) and obese (38.7%) participants compared to those with normal weight (24.9%, Supplementary Tables 4, 5).

**Figure 6:**
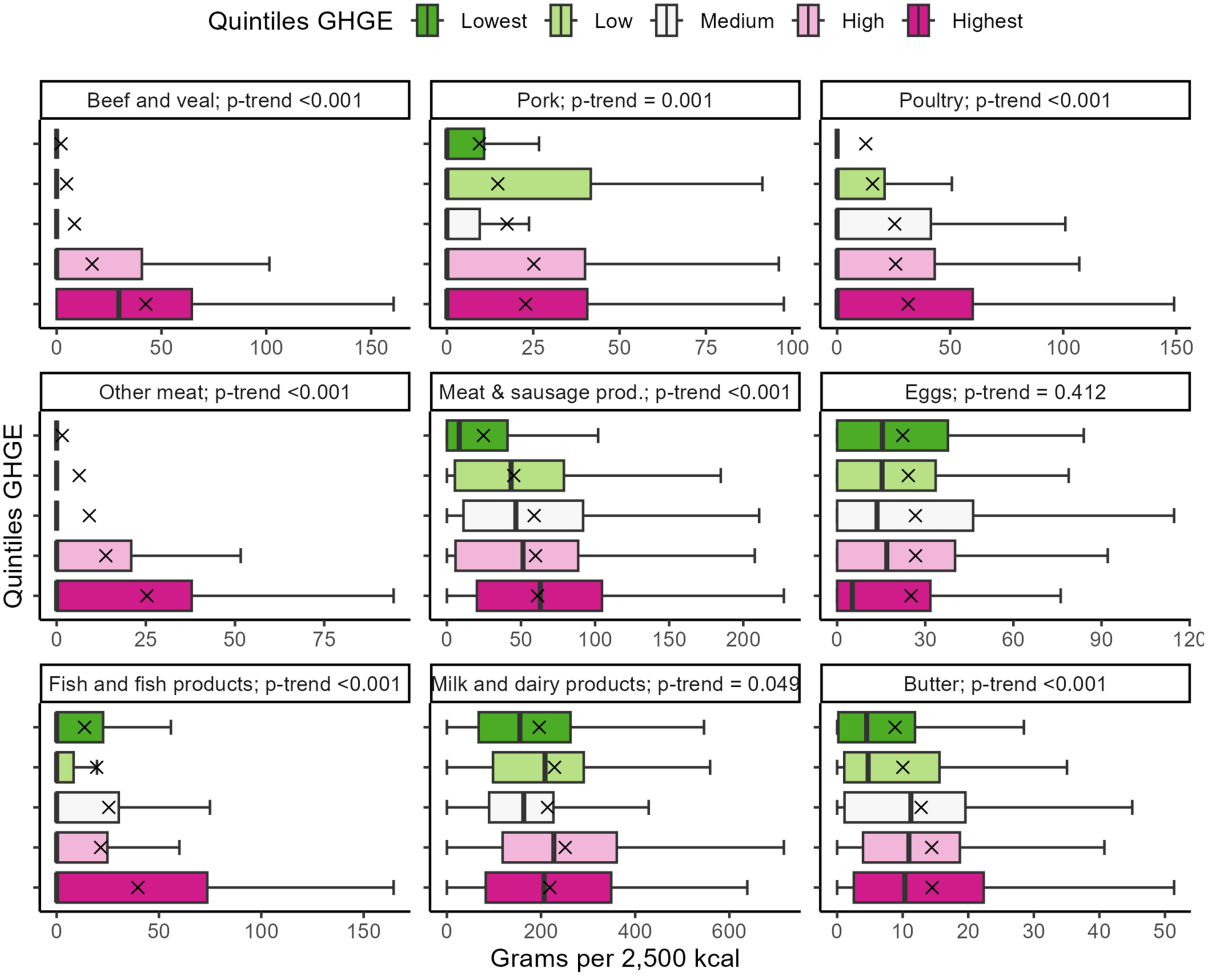
Consumption of foods of animal origin across the GHGE quintiles (*N* = 1,100). Intakes were adjusted to 2,500 kcal. Crosses depict the means. Whiskers extend from the 5th to the 95th percentile. Outliers were omitted from visual depiction only. Data are weighted to represent the Bavarian population. GHGE, greenhouse gas emissions.

**Figure 7:**
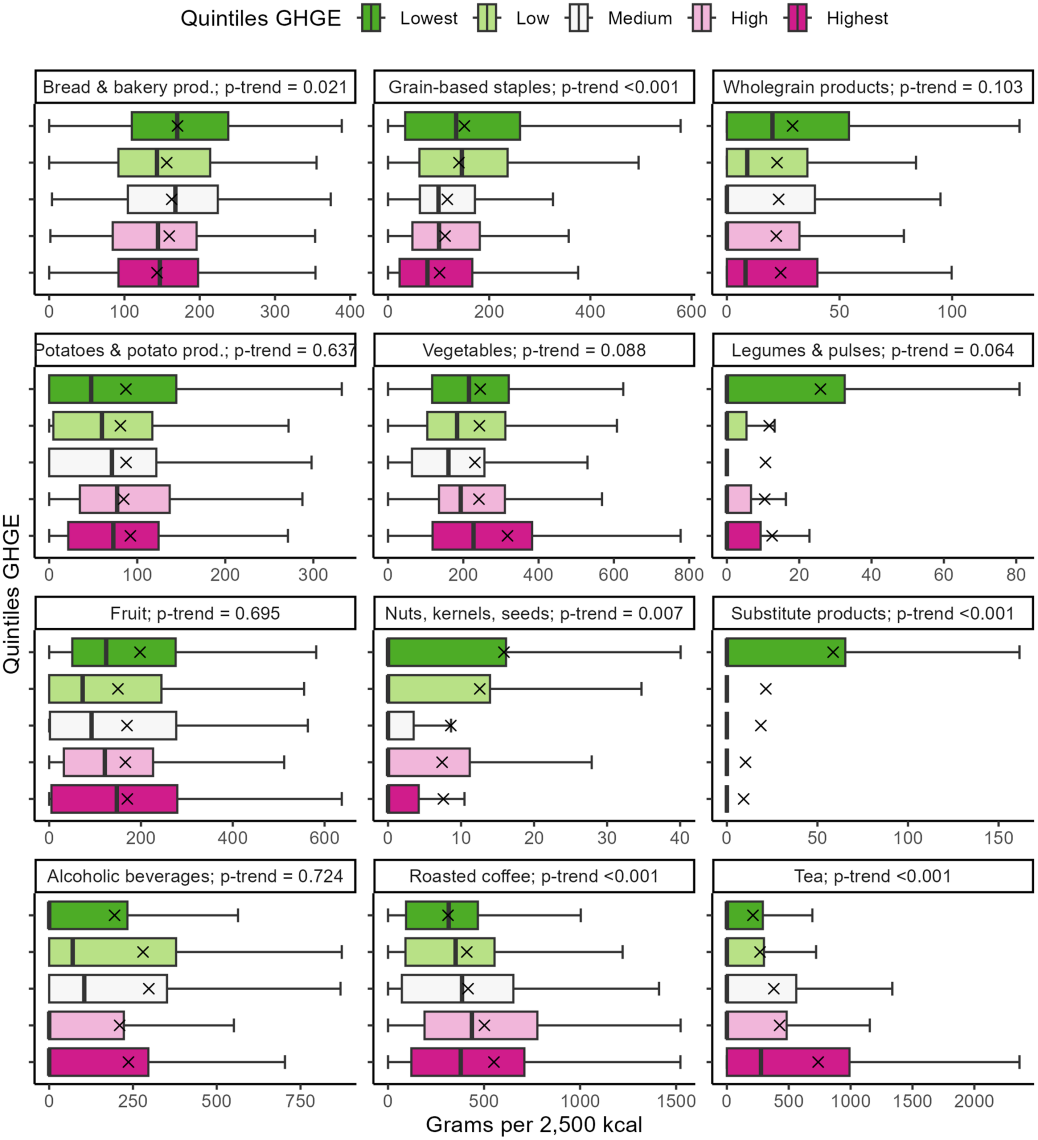
Consumption of plant-based foods and beverages across the GHGE quintiles (*N* = 1,100). Intakes were adjusted to 2,500 kcal. Crosses depict the means. Whiskers extend from the 5th to the 95th percentile. Outliers were omitted from visual depiction only. Data are weighted to represent the Bavarian population. GHGE, greenhouse gas emissions.

Visual depictions of the food and beverage consumption adjusted to 2,500 kcal across the GHGE quintiles are presented in Figure 6 for foods of animal origin and in Figure 7 for plant-based foods and beverages. High emitters consumed more foods of animal origin, including butter, fish and fish products, meat and sausage products, meat, i.e., beef and veal, pork, poultry, and other meat (all *p*-trend ≤0.001), as well as milk and dairy products (*p*-trend = 0.049). They also had a noticeable higher intake of roasted coffee and tea and other infusion (all *p*-trend <0.001). Low emitters consumed significantly more grain-based staple foods, substitute products, nuts, kernels, and seeds (*p*-trend ≤0.007), and indicatively more legumes and pulses (*p*-trend = 0.064). Alcoholic and non-alcoholic beverages, eggs, fruit, potatoes, vegetables, and whole-grain products showed no significant trends (*p*-trend ≥0.140). A detailed tabular depiction of all food group intakes can be seen in Supplementary Table 6.

## 4 Discussion

### 4.1 Response

While modest, the BVS III’s overall response rate of 26.0% reflects both the anticipated challenges of modern survey research and careful planning. While the response rate of the predecessor study BVS II in 2002/2003 was substantially higher with 51.3% (70.9% of cleaned gross sample) (42), low response rates are typical in contemporary survey environments (43–46). Postema et al. (46) described that the response rate in the PIENTER studies, three nationwide serological surveys in the Netherlands, declined from 55.0% in PIENTER 1 (1995/1996) to 33.9% in PIENTER 2 (2006/2007) to 15.8% in the most recent PIENTER 3 (2016/2017). Similarly, the response in the National Maternity Surveys in England dropped from 67% in 1995 to 29% in 2018. Definite reasons for the decline in response rates remain unclear. However, a variety of factors are deemed important, including survey fatigue (particularly during the COVID-19 pandemic) (47, 48), privacy concerns, topic sensitivity (49), participation effort, and the logistical challenges of reaching diverse population groups. These factors often introduce non-response biases, skewing the study sample toward specific characteristics. The BVS III participants reflect this common profile seen in many surveys and studies, typically being more often female, older, married, and higher educated (43, 50).

As these trends are anticipated challenges of modern survey research, they necessitate preventive or corrective measures. Accordingly, more addresses from the residents’ registration offices were requested for subject acquisition, ensuring that the final sample size would reach the targeted 1,500 participants even with anticipated dropouts. This approach resulted in a final sample of 1,503 participants, providing a solid basis for the study’s conclusions. Moreover, the use of weighting factors helped to counterbalance non-response biases, making the findings more reflective of the general Bavarian population.

The COVID-19 pandemic introduced additional challenges before and during the survey’s field phase. Notably, 718 individuals cited quarantine as their reason for non-participation, the largest share of the QNDs. This may reflect both the genuine impact of the pandemic and the possibility that some potential subjects might have used the pandemic as a convenient excuse to opt out. Albeit COVID-19 quarantine was categorized as QND, this dropout might have disproportionately affected specific groups. Within this study, the reason for non-participation was not further analyzed. To the authors’ knowledge, there is no evidence in the literature suggesting that COVID-19-related measures, i.e., quarantine or “lockdown,” were wrongfully used as justification for non-participation in other studies. Gao et al. reported in 2023 that COVID-19 pandemic-related variables (i.e., previous COVID-19 infection or job loss due to the COVID-19 pandemic) positively correlated with the dropout rate among psychosomatic rehabilitation patients in a web-based study (51). Krieger et al. compared the response rates of six major surveys in the US before and during the onset of the COVID-19 pandemic. All but one ongoing surveys reported a relative decline in response rates of approximately 29%, with the largest declines seen among individuals with lower income and education levels (52).

### 4.2 Dietary sustainability

This work used established databases that provided sustainability metrics for the foods consumed in the BVS III. The choice of underlying databases influences environmental sustainability estimates. LCA methodologies differ and include different system boundaries, data sources, and assumptions. For example, the SHARP-ID and SuEATableLife databases, while both providing valuable insights, differ in their scope and approach. SHARP-ID gives estimates on dietary GHGE and LU based on EU-averages and encompasses the entire life cycle of food products, beginning with primary production and includes processing, packaging, transport, and post-market processing (36). However, the use of EU-averages in SHARP-ID means that the environmental impacts of imported foods, such as beef from non-EU countries, are not fully captured, potentially underestimating the footprint of diets reliant on such imports. In contrast, the SuEATableLife database was used to assess the WFP in this work. SuEATableLife also gives estimates for GHGE, which were not used in this work. System boundaries extend only to the distribution center, excluding post-market processing such as cooking (37). These differences lead to variations in environmental impact estimates. This was highlighted by Carvalho et al. by comparing commonly used dietary sustainability databases, i.e., SHARP-ID (36), SuEATableLife (37), and values provided by Poore and Nemecek (53). Same food consumption data were linked to these databases and dietary GHGE were compared, which led to notable discrepancies in GHGE estimates with medians of 4.42 kg CO_2_eq for SHARP-ID, 5.64 kg CO_2_eq for SuEATableLife, and 6.17 kg CO_2_eq for Carvalho et al. (54). These variations reflect the importance of carefully selecting databases for dietary sustainability assessments. The highlighted differences in system boundaries, data sources, and overall purpose of selected database significantly influence results (12). For instance, as SHARP-ID uses EU-averages (36), it is suitable for intra-EU comparisons, while global databases like Poore and Nemecek (53) serve broader, potentially cross-regional intent. Additionally, SuEATableLife provides WFP data but does not differentiate between the type of water usage (37), i.e., green, blue, and gray water (55), which may limit its applicability for certain analyses. SHARP-ID was chosen in this study to ensure comparability within the EU and to align with other, EU-based research. SuEATableLife was selected because it provides comprehensive WFP data for a wide range of food items to complement GHGE and LU estimates by SHARP-ID.

In this study, the sustainability metrics were standardized to a fixed energy intake of 2,500 kcal. Also, emission groups were defined based on quintiles of the standardized GHGE per 2,500 kcal. These methodological choices allowed for an energy-independent comparison of dietary environmental impact of the dietary composition rather than absolute food consumption. In contrast, assessing GHGE per day without adjustment reflects the total environmental burden of an individual’s diet but may be influenced by variations in energy intake and is susceptible to known biases in dietary surveys based on 24-h recalls, such as social desirability bias (56) or recall bias (57), where participants consciously or unconsciously over- or underreport their dietary intakes. By standardizing GHGE to 2,500 kcal, we aimed to mitigate the influence of these biases and provide a more accurate comparison of dietary sustainability across participants. When using GHGE per day, individuals with a high total energy intake, possibly due to high energy requirements, may be placed in higher GHGE groups, even if their diet has a relatively low environmental impact when standardized. Conversely, while standardizing to 2,500 kcal accounts for differences in energy intake and focuses on the dietary composition itself, it can, however, lead to shifts in the classification. Participants consuming large amounts of foods with low energy-density or having an overall low caloric intake could possibly have low GHGE per day but may be placed in higher GHGE groups per 2,500 kcal. While the food group contribution to GHGE differed only marginally between the energy-adjusted versus unadjusted/per day approach, these methodological differences highlight the importance of carefully interpreting results and comparing studies. These methodological considerations align with findings from a recent study, which demonstrated a strong correlation between energy intake and GHGE per day. The study also showed that the assessment of micronutrient adequacy varied depending on whether energy intake was standardized. However, micronutrient intake was not examined in our study, and our methodological approach remains valid within its intended framework. Rather, these findings underscore the importance of carefully considering whether energy standardization is appropriate based on the specific research question (58). Several studies have applied similar energy standardization when assessing the environmental impact of diets, enabling more consistent cross-study comparisons (15, 17, 19). The EAT-Lancet Commission provided dietary recommendations based on an intake of 2,500 kcal per day, known as the Planetary Health Diet (1). Standardizing GHGE to 2,500 kcal allowed for direct comparisons with this benchmark while controlling for variations in energy intake.

In 2012, the FAO emphasized assessing diets for environmental sustainability and nutritional value, defining sustainable diets as those minimizing environmental impact, supporting food security, and promoting health for current and future generations (59). It is now generally accepted that sustainable nutrition should not only encompass the environmental impact but also be nutritionally adequate, socially viable, and economically performative (60–62). In line with this, the EAT-Lancet Commission (1) provided the Planetary Health Diet for an intake of 2,500 kcal/d, which integrated all facets of sustainable diets to facilitate sustainable food systems by 2050 (1). Based on this framework, the assumption of a global population of 10 billion people in 2050 and a GHGE boundary for the food production of 5 Gt CO_2_eq per year (1), dietary GHGE should not exceed 1.37 kg CO_2_eq per person and day with an intake of 2,500 kcal. The average dietary GHGE in Bavaria amounts to 6.14 kg CO_2_eq per 2,500 kcal, which is approximately 4.5 times higher than the defined boundary by the EAT-Lancet Commission. The lowest GHGE quintile in Bavaria still emitted 4.24 kg CO_2_eq per 2,500 kcal, exceeding the planetary boundary more than three times. This considerable over-emission can be largely attributed to the high consumption of animal-derived foods, which accounted for almost two-thirds of dietary GHGE in the BVS III. These products were disproportionately consumed in higher amounts among high emitters. Due to their high environmental impact and potentially health-impairing effects, animal-derived foods are not emphasized in the Planetary Health Diet (1). Conversely, foods emphasized in the Planetary Health Diet, such as legumes, nuts, and potatoes (1), were not considerably consumed overall, except for legumes and meat substitutes in the lowest emitter group. Substitute products for meat and dairy were also not considerably consumed. Due to the low consumption, these foods had little to no contribution to GHGE. This disparity highlights the need for dietary shifts toward more sustainable food choices to align with planetary health goals.

Comparable data in Germany are limited. Koelman et al. (19) reported notably higher dietary emissions compared to the BVS III of 6.6 kg CO_2_eq for men and 7.0 kg CO_2_eq per 2,000 kcal in an Eastern German population, which extrapolates to 8.25 and 8.75 kg CO_2_eq per 2,500 kcal for men and women, respectively. Comparably, women in the BVS III emitted 6.11 kg CO_2_eq per 2,500 kcal, while men’s emissions were 6.17 kg CO_2_eq. However, the study’s design and population, primarily aged 35–65 years, limit comparisons. The study population was comprised of a subsample of the EPIC (1994–1998) study cohort members who were still actively participating in the follow-up (2010–2012). It relied on FFQs to assess dietary intake, unlike the 24-h dietary recalls used in the BVS III (19).

Similarly, the Bavarian dietary GHGE of 6.14 kg CO_2_eq per 2,500 kcal and LU of 7.50 m^2^yr per 2,500 kcal were generally comparable to those reported in other European studies by Mertens et al. (17). Mertens et al. (17) energy-adjusted GHGE and LU values to 2,000 kcal. These values were projected to be 2,500 kcal for comparison. Bavarian food-related emissions resembled those in Denmark (6.25 kg CO_2_eq and LU of 7.88 m^2^*yr. per 2,500 kcal) and Italy (6.13 kg CO_2_eq and LU of 7.88 m^2^*yr. per 2,500 kcal) while superseding those in the Czech Republic (5.5 kg CO_2_eq and LU of 7.13 m^2^*yr. per 2,500 kcal). France reported the highest values, with an average GHGE of 8.0 kg CO_2_eq and LU of 10.0 m^2^*yr. per 2,500 kcal. Again, these comparisons are constrained by differences in study populations, design, and data collection periods (2003–2008) (17). Dietary data were, however, linked to the same sustainability metric database, SHARP-ID, for all studies mentioned.

The current study found that high emitters per 2,500 kcal were more likely to be female, indicatively older, and have a higher BMI or waist circumference, which aligns with findings from Mertens et al. (17), where energy-adjusted GHGE increased with age in Denmark and France. Additionally, women in the Czech Republic and France exhibit higher GHGE, supporting the depicted sex differences. Also, Koelman et al. (19) reported higher energy-adjusted GHGE in women and with higher BMI. The current study found no significant effect of education when controlling for other factors. This was confirmed to a certain extent: Education levels positively correlated with GHGE in the Czech Republic (17) and Northern Sweden (15) but negatively correlated in France (17). However, sex-specific differences in dietary sustainability metrics are controversially discussed. While the sex-specific GHGE and WFP increases in females were highly significant in this study (*p* ≤ 0.023), the regression models offered low explanation of variance in dietary GHGE (adjusted *R*^2^ = 0.10) and WFP (adjusted *R*^2^ = 0.08). This indicates that the statistically significant sex differences explained only a modest proportion of the variance in dietary GHGE and WFP. Other factors not included in the models may contribute substantially to the observed variability. However, other studies reported an inverse relationship. Exemplarily, men showed higher energy-adjusted GHGE in the USA (63). Likewise, in a young Spanish study population, females consumed diets with lower GHGE density significantly more often (64). Against this, Koelman et al. found higher energy-adjusted emissions for Eastern German women (19). Here, by adding the food groups “tea and other infusion,” “roasted coffee” and “milk and dairy products” to the regression analysis as additional predictors, men and women no longer differed in terms of CO_2_eq emissions (*β* = 0.022, *p* = 0.827) (data not shown). This suggests that the previously observed sex-specific differences in CO2eq emissions might be driven primarily by differences in the consumption patterns of these specific high-impact food groups and can largely be attributed to the differences in the consumption of these food groups. Likewise, the sex-specific differences in the WFP were reduced but not entirely resolved by controlling for these food groups (*β* = 0.159, *p* = 0.001) (data not shown). This indicates that while these food groups play a significant role in explaining sex-specific differences in WFP, other food groups or factors may also contribute to the remaining variance. Rippin et al. (14) analyzed the GHGE of individual diets in the UK and found that beverages, particularly tea, coffee, and alcoholic drinks, were major contributors. These items, along with cakes, biscuits, and confectionery, accounted for a quarter of the diet-related GHGE. The authors suggested that targeting these food and drink categories could offer alternative strategies for reducing diet-related GHGE, which is supported by the results depicted in this study. Despite limited information on the direct impact of coffee and tea on dietary GHGE at the individual level, the issue has been widely discussed (65–68). As tropical and subtropical plants, coffee and tea are commonly grown in monocultures, negatively affecting biodiversity (68) and fostering deforestation, erosion, and river pollution (69–71) in these regions. The high reported consumption of these beverages in Bavaria, especially in the high and highest GHGE quintiles, consequently, exerts additional environmental stress. Largely, previous studies lack a comprehensive analysis of the LU, GHGE, and WFP associated with the consumption of coffee and tea (14, 67), leaving gaps in understanding their full environmental effects, and underscoring the need for further research on their ecological and climate-related effects. Furthermore, the positive association between waist circumference and GHGE observed in the regression model (*β* = 0.01, *p* < 0.001) was most probably driven by the disproportionately high contribution of meat, sausages, and meat products to dietary emissions in pre-obese and obese (Supplementary Tables 4, 5). This is consistent with additional data from the BVS III, which showed that obese and pre-obese individuals consumed larger quantities of meat compared to those with normal weight, regardless of energy-adjustment (data not shown). This is in line with a plethora of publication that associated higher BMI with greater meat consumption, particularly red and processed meats (72–74), which was also the case in the BVS III (data not shown). In line with the findings from the BVS III, Strid et al. (15) showed in a Northern Swedish population that pre-obese and obese participants had the highest energy-adjusted GHGE. Energy was, however, adjusted to 1,000 kcal.

Sustainable food choices are determined by a complex interplay between individual perceptions of the environmental impact of foods and their actual behaviors. The results of this study aligned with broader research that highlighted discrepancies between individuals’ awareness of the environmental impact of dietary choices, their intentions, and actual behaviors (25, 75). Notably, low emitters in this study demonstrated a more accurate perception of certain practices. For instance, they perceived the environmental impact of seasonal fruit and vegetable consumption as lower, while higher emitters tended to attribute a higher significance to this behavior. Additionally, low emitters in the study showed a greater awareness of the environmental impact of reduced meat consumption, which was also reflected in their behavior, indicating a closer alignment between perceptions and effective behaviors related to sustainability. This is consistent with research showing that consumers often overestimated the benefits of regional and seasonal products while simultaneously underestimating the impact of the reduction of meat (76, 77) and dairy consumption (78). Although higher emitters expressed intentions to engage in sustainable practices, their assessment of the relative impact of these habits appeared less accurate. These findings underscore the importance of enhancing consumer education regarding the environmental consequences of dietary choices to promote more sustainable eating behaviors.

### 4.3 Limitations

The BVS III was conducted partly during the COVID-19 pandemic, which potentially affected food availability and dietary behaviors. The unique circumstances of the pandemic, including lockdowns and changes in food supply chains, should be considered when interpreting the results. While the reliance on 24-h dietary recalls is prone to reporting bias, this limitation was mitigated by employing trained interviewers to conduct the recalls with participants, computation of the individual recalls into weighted averages to account for weekday-specific changes in the diet, omitting of extreme underreporters, and energy-adjusted data analyses. The survey employed a robust methodology, incorporating strategic oversampling and weighting factors to adjust for demographic imbalances and account for deviations from the underlying population, resulting in data closely aligned with Bavarian population characteristics. Nonetheless, the BVS III achieved a response rate of 26.0%, reflecting the broader trends in declining survey participation rates observed globally. The sustainability databases SHARP-ID and SuEATable Life were used in this work. The calculated dietary greenhouse gas emissions and resource use are highly dependent on the database used.

### 4.4 Conclusion

This study assessed the dietary GHGE, LU, and WFP in Bavaria, identifying significant differences across demographic and dietary intake groups. The average GHGE was 6.14 kg CO_2_eq, LU was 7.50 m^2^*yr., and WFP was 4.39 kiloliters per 2,500 kcal. According to the Planetary Health Diet framework from the EAT-Lancet Commission (1), the average Bavarian exceeded the recommended GHGE boundary by 4.5 times, with even the lowest GHGE quintile surpassing it threefold, indicating an urgent call for action. Foods of animal origin have expectedly emerged as significant drivers of dietary GHGE. This, on the one hand, underscored the need to address their ecological impact, especially concerning the persistent underestimation of meat consumption on dietary GHGE and resource use among the highest emitters. Low emitters had a more accurate perception of the environmental benefits of reducing meat consumption, and their behavior aligned with this awareness, as a larger proportion actively reduced their meat intake. On the other hand, provision of information alone is unlike to drive meaningful behavior change, as education showed no association with GHGE when controlling for confounders. To support ecological sustainability and public acceptance, meat consumption should be critically addressed, realistically categorized, but not stigmatized or restricted. In addition, more sustainable food options, such as plant-based substitutes, or dietary behaviors, such as meat reduced diets (“flexitarian”) or meat consumption only for special occasions or festivities, should be made more attractive, accessible, and socially acceptable. Along with this, improving the quality of meat by improving the quality of livestock farming should be further pursued. These are all to a small extent the sole responsibility of the individual. Rather, it is the responsibility of the governing authorities to create framework conditions through financial support or incentives that ease sustainable food choices and improve the food environment. In this work, the equalized net household income had no association with GHGE. Creating financial incentives therefore does not exacerbate existing fiscal inequalities and could be a possible lever. Beyond products of animal origin, high emitters notably consumed more coffee and tea and other infusions, suggesting they may be overlooked contributors to dietary GHGE and resource use. Higher emissions per 2,500 kcal were associated with higher BMI or waist circumference, being female, and following an omnivorous diet. These findings point to a need for more nuanced discourse that addresses sustainable dietary shifts across relevant demographic groups with more targeted approaches.

## Data Availability

The datasets presented in this article are not readily available because the data that support the findings of this study will be available following the publication of the primary results in the coming months. Afterward, they can be requested upon reasonable justification, including a description of the intended analysis and approval from the project partners. All requests must ensure proper use, with any misuse strictly prohibited. Requests to access the datasets should be directed to kgedrich@tum.de.
