# Supplementary material for: Dietary greenhouse gas emissions and resource use among Bavarian adults: associations with sociodemographics and food choices
Source: Front Nutr. 2025 Apr 9;12:1542254. doi: 10.3389/fnut.2025.1542254 (PMC12014458; doi:10.3389/fnut.2025.1542254)
Supplement: Supplementary file 1 [file Table_1.docx]

Supplementary Material

# Supplementary Methods

**Study Design**

The BVS III comprised multiple components. First, the participants were visited at home. The home visit consisted of a standardized and interviewer-led computer-assisted personal interview (CAPI), followed by a self-administered computer-assisted self-interview (CASI), including questions regarding body mass and height. Further, waist circumference and blood sugar levels were measured during the visit, and dried bloodspot (DBS) samples were taken to assess HbA1c and total cholesterol. The interviewers guided the participants in performing the sample collection. While fasting status was not mandatory, it was documented if applicable. The questionnaires used in CAPI and CASI encompassed inquiries regarding various aspects of dietary behavior, such as general eating habits, eating out or communal eating practices, and levels of nutritional knowledge, education, and communication. Additionally, they addressed tobacco consumption, medical history and demographic information. Sustainability was another focal point, with a dedicated section of questions. The selection of questions was based on various publications (1-3) and mainly on Tobler et al. (2). Given the context of the COVID-19 pandemic during the study, supplementary questions were included concerning COVID-19 and its impact. Moreover, female participants were asked additional questions, including aspects like pregnancies and breastfeeding behaviors. Particularly, the following validated questionnaires were part of the home visit:

- Single-Item Eating Motives (4) based on The Eating Motivation Survey (TEMS) (5)
- Alcohol Use Disorders Identification Test-C (AUDIT-C) (6)
- Veterans RAND 12-item health-related quality of life questionnaire (VR-12) (7) based on the Veterans Health Study (8)
- Physical activity according to the European Health Interview Survey - Physical Activity Questionnaire (EHIS-PAQ) (9) and calculation of physical activity according to Gerrior et al. (10) based on daily commuting activity (i.e., walking and cycling), work effort, and sports.
- Respiratory tract infection (RTI) score to indicate susceptibility to respiratory tract infections according to the respiratory tract infection susceptibility study (AWIS; German: *Atemswegsinfektanfälligkeits-Studie*) (11)

Following the initial home visits, participants underwent repeated telephone interviews over the subsequent six weeks to document their food consumption, including dietary supplements, over the past day. The recorded days were randomly selected and should record dietary intake during two weekdays and one weekend day. These 24-h diet recalls were facilitated through computer-assisted telephone interviews (CATI) utilizing GloboDiet©, a computer-based survey program derived from EPIC-SOFT© and developed by the International Agency for Research on Cancer (Lyon, France). The Max Rubner-Institute (Karlsruhe, Germany) provided the German version. It was intended to obtain three dietary recalls per participant. During the home visits, participants were provided with a photo book to aid in estimating consumed quantities. This 110-page book allowed participants to reference different foods and dishes in various portion sizes.

The market research company KANTAR (Munich, Germany) was commissioned to collected the data. The DBS samples were sent to the external analysis laboratory Vitas AS (Oslo, Norway) to analyze HbA1c and total cholesterol. Vitas AS also provided the DBS kits for sample collection. The home visits were carried out from October 2021 to November 2022. The dietary 24-h recalls were conducted from October 2021 to January 2023.

# Supplementary Results

**Response**

Out of the 7,449 individuals contacted, a total of 1,503 participated in the CAPI/CASI via house visits. For 1,239 participants, at least one 24-h recall was recorded. Valid dietary data were recorded for 1,100 participants, excluding participants with less than two 24-h recalls and underreporters. Some variables had missing data points. The sample sizes for each variable were as follows: waist circumference (N = 1,448), education (N = 1,502), equivalized net household income (N = 1,380), civil status (N = 1,501), employment (N = 1,502), and smoking status (N = 1,502). Absolute numbers may differ when weighting is applied

Out of the 7,449 people contacted, 1,679 (22.5%) were classified as quality-neutral dropouts (QND). These were divided into address-related QNDs (582 cases or 7.8%) and other QNDs (1,097 cases or 14.7%). Among the address-related QNDs, 53 cases (9.1%) involved non-existent addresses, 87 individuals (14.9%) had relocated within Germany, 46 (7.9%) had moved abroad, while the majority, 396 individuals (68.0%), had moved to unknown addresses. The other QNDs included 10 deceased individuals (0.9%), 28 not residing in private households (2.6%), 341 with insufficient German language skills (31.1%), and 718 who reported to be in COVID-19-related quarantine at the time of the survey (65.4%).

The cleaned gross sample, excluding QNDs, comprised 5,770 individuals, 77.5% of the total sample. 3,056 (53.0% of the cleaned gross sample) refused to participate in the study. The interviewers did not contact another 284 cases (4.9%) because the target number of the respective sample points was met. In 514 cases (8.9%), the target person could not be reached at the household, and in 369 cases (6.4%), no appointment could be scheduled within the specified field period. 44 target individuals were unable to participate due to self-reported health issues (Supplementary Table 7).

1,503 participants, corresponding to 26.0% response proportion, were interviewed during the home visit and completed the CAPI/CASI questionnaires. Of these, waist circumference was measured for 1,448 participants, blood sugar levels for 1,346 participants, HbA1c for 1,294 participants, and total cholesterol for 1,260 participants.

Only participants with at least two 24-h recalls were considered for the estimation of dietary intake. This applied to 1,148 or 76.4% of participants. Of these, 48 people were excluded as underreporters, resulting in a final sample size for the dietary assessment of 1,100 participants. 17.6 % of the participants who completed the house visit were not reached for the dietary assessment (Supplementary Table 8).

# Supplementary Tables

Supplementary Table 1 Description of the BVS III study sample with at least two 24-h recalls (excluding underreporters; N = 1,100)

|  |  |  | **Sex** | |  |
| --- | --- | --- | --- | --- | --- |
| **Variable** | **N** | **Overall**, N = 1100 (100%)*^1^* | **Male**, N = 485 (44%)*^1^* | **Female**, N = 615 (56%)*^1^* | **p-value***^2^* |
| **Age (in years)** | 1,100 | 49.3 ± 14.9 | 49.3 ± 14.8 | 49.3 ± 14.9 | >0.9 |
| **Age group (in years)** | 1,100 |  |  |  | >0.9 |
| 18-24 |  | 71 (6%) | 30 (6%) | 41 (7%) |  |
| 25-34 |  | 156 (14%) | 71 (15%) | 85 (14%) |  |
| 35-50 |  | 297 (27%) | 126 (26%) | 171 (28%) |  |
| 51-64 |  | 387 (35%) | 176 (36%) | 211 (34%) |  |
| ≥65 |  | 189 (17%) | 82 (17%) | 107 (17%) |  |
| **BMI (in kg/m^2^)** | 1,100 | 25.8 ± 4.8 | 26.9 ± 4.3 | 25.0 ± 5.1 | <0.001 |
| **BMI group***^3^* | 1,100 |  |  |  | <0.001 |
| Underweight |  | 23 (2%) | 3 (1%) | 20 (3%) |  |
| Normal weight |  | 520 (47%) | 180 (37%) | 340 (55%) |  |
| Pre-obesity |  | 367 (33%) | 197 (41%) | 170 (28%) |  |
| Obesity |  | 190 (17%) | 105 (22%) | 85 (14%) |  |
| **Waist circumference (in cm)** | 1,073 | 93.1 ± 15.0 | 100.3 ± 13.2 | 87.3 ± 13.8 | <0.001 |
| **Education** | 1,100 |  |  |  | 0.010 |
| Low |  | 220 (20%) | 98 (20%) | 122 (20%) |  |
| Middle |  | 317 (29%) | 118 (24%) | 199 (32%) |  |
| High |  | 563 (51%) | 269 (55%) | 294 (48%) |  |
| **Equivalized net household income (in Euro/month)** | 1,021 | 2,074.2 ± 1,463.7 | 2,044.6 ± 1,426.3 | 2,097.0 ± 1,492.8 | 0.812 |
| **Civil status** | 1,099 |  |  |  | <0.001 |
| Single |  | 148 (13%) | 72 (15%) | 76 (12%) |  |
| Unmarried - in a partnership |  | 146 (13%) | 65 (13%) | 81 (13%) |  |
| Married |  | 697 (63%) | 321 (66%) | 376 (61%) |  |
| Widowed |  | 29 (3%) | 4 (1%) | 25 (4%) |  |
| Divorced |  | 79 (7%) | 23 (5%) | 56 (9%) |  |
| **Living situation** | 1,100 |  |  |  | 0.335 |
| Living alone in a private household |  | 171 (16%) | 67 (14%) | 104 (17%) |  |
| Living in a private household with family/friends or other persons |  | 926 (84%) | 417 (86%) | 509 (83%) |  |
| Other |  | 3 (0%) | 1 (0%) | 2 (0%) |  |
| **Employment** | 1,100 |  |  |  | 0.002 |
| Employed |  | 705 (64%) | 326 (67%) | 379 (62%) |  |
| Marginally, occasionally or irregularly employed |  | 29 (3%) | 4 (1%) | 25 (4%) |  |
| In vocational training/apprenticeship/retraining |  | 18 (2%) | 11 (2%) | 7 (1%) |  |
| Currently not employed: unemployed or job-seeking, on parental leave |  | 59 (5%) | 18 (4%) | 41 (7%) |  |
| Retired, pensioner, homemaker |  | 246 (22%) | 105 (22%) | 141 (23%) |  |
| Other (e.g., pupil, student, assisting family member) |  | 43 (4%) | 21 (4%) | 22 (4%) |  |
| **Smoking** | 1,099 |  |  |  | 0.011 |
| Never |  | 587 (53%) | 239 (49%) | 348 (57%) |  |
| Currently |  | 172 (16%) | 92 (19%) | 80 (13%) |  |
| In the past |  | 340 (31%) | 153 (32%) | 187 (30%) |  |
| **Physical activity group***^4^* | 1,100 |  |  |  | <0.001 |
| Sedentary |  | 294 (27%) | 102 (21%) | 192 (31%) |  |
| Low active |  | 298 (27%) | 118 (24%) | 180 (29%) |  |
| Active |  | 250 (23%) | 113 (23%) | 137 (22%) |  |
| Very active |  | 258 (23%) | 152 (31%) | 106 (17%) |  |
| **Diet type** | 1,100 |  |  |  | 0.002 |
| Omnivorous |  | 1,009 (92%) | 459 (95%) | 550 (89%) |  |
| Vegetarian or vegan |  | 91 (8%) | 26 (5%) | 65 (11%) |  |
| *^1^*Mean ± SD; n (%) | | | | | |
| *^2^*Wilcoxon rank sum test; Kruskal-Wallis rank-sum test; Pearson's Chi-squared test; Fisher's exact test | | | | | |
| *^3^*According to the definition of the WHO (12) | | | | | |
| *^4^*According to Gerrior et al. (10) | | | | | |

Supplementary Table 2 Demographic characteristics in Bavaria. Data are weighted to represent the Bavarian population. Based on the BVS III study sample with at least two 24h-recalls (N = 1,100).

|  |  |  | **Sex** | |  |
| --- | --- | --- | --- | --- | --- |
| **Variable** | **N** | **Overall**, N = 1100 (100%)*^1^* | **Male**, N = 558 (51%)*^1^* | **Female**, N = 542 (49%)*^1^* | **p-value***^2^* |
| **Age (in years)** | 1,100 | 46.7 ± 0.8 | 46.4 ± 1.1 | 47.1 ± 1.1 | 0.587 |
| **Age group (in years)** | 1,100 |  |  |  | >0.9 |
| 18-24 |  | 105 (10%) | 52 (9%) | 52 (10%) |  |
| 25-34 |  | 202 (18%) | 109 (20%) | 93 (17%) |  |
| 35-50 |  | 311 (28%) | 160 (29%) | 151 (28%) |  |
| 51-64 |  | 317 (29%) | 158 (28%) | 159 (29%) |  |
| ≥65 |  | 165 (15%) | 79 (14%) | 86 (16%) |  |
| **BMI (in kg/m^2^)** | 1,100 | 26.0 ± 0.3 | 26.8 ± 0.3 | 25.3 ± 0.4 | <0.001 |
| **BMI group***^3^* | 1,100 |  |  |  | <0.001 |
| Underweight |  | 18 (2%) | 1 (0%) | 17 (3%) |  |
| Normal weight |  | 514 (47%) | 201 (36%) | 313 (58%) |  |
| Pre-obesity |  | 362 (33%) | 244 (44%) | 118 (22%) |  |
| Obesity |  | 206 (19%) | 112 (20%) | 94 (17%) |  |
| **Waist circumference (in cm)** | 1,061 | 93.9 ± 0.8 | 99.8 ± 1.0 | 87.5 ± 1.1 | <0.001 |
| **Education** | 1,100 |  |  |  | 0.464 |
| Low |  | 365 (33%) | 194 (35%) | 171 (32%) |  |
| Middle |  | 322 (29%) | 148 (26%) | 174 (32%) |  |
| High |  | 413 (38%) | 217 (39%) | 197 (36%) |  |
| **Equivalized net household income (in Euro/month)** | 1,018 | 2,089.0 ± 75.0 | 2,031.8 ± 107.3 | 2,146.1 ± 104.3 | 0.658 |
| **Civil status** | 1,100 |  |  |  | 0.211 |
| Single |  | 160 (15%) | 93 (17%) | 67 (12%) |  |
| Unmarried - in a partnership |  | 193 (18%) | 89 (16%) | 103 (19%) |  |
| Married |  | 659 (60%) | 343 (61%) | 316 (58%) |  |
| Widowed |  | 32 (3%) | 7 (1%) | 25 (5%) |  |
| Divorced |  | 56 (5%) | 25 (5%) | 31 (6%) |  |
| **Living situation** | 1,100 |  |  |  | 0.592 |
| Living alone in a private household |  | 179 (16%) | 84 (15%) | 95 (17%) |  |
| Living in a private household with family/friends or other persons |  | 920 (84%) | 473 (85%) | 447 (82%) |  |
| Community-oriented living arrangement |  | 0 (0%) | 0 (0%) | 0 (0%) |  |
| Other |  | 1 (0%) | 1 (0%) | 0 (0%) |  |
| **Employment** | 1,100 |  |  |  | 0.061 |
| Employed |  | 702 (64%) | 375 (67%) | 326 (60%) |  |
| Marginally, occasionally or irregularly employed |  | 29 (3%) | 5 (1%) | 24 (4%) |  |
| In vocational training/apprenticeship/retraining |  | 26 (2%) | 15 (3%) | 12 (2%) |  |
| Currently not employed: unemployed or job-seeking, on parental leave |  | 58 (5%) | 18 (3%) | 40 (7%) |  |
| Retired, pensioner, homemaker |  | 228 (21%) | 108 (19%) | 120 (22%) |  |
| Other (e.g., pupil, student, assisting family member) |  | 57 (5%) | 38 (7%) | 20 (4%) |  |
| **Smoking** | 1,099 |  |  |  | 0.301 |
| Never |  | 554 (50%) | 271 (49%) | 283 (52%) |  |
| Currently |  | 184 (17%) | 109 (20%) | 75 (14%) |  |
| In the past |  | 362 (33%) | 177 (32%) | 184 (34%) |  |
| **Physical activity group***^4^* | 1,100 |  |  |  | <0.001 |
| Sedentary |  | 258 (23%) | 97 (17%) | 162 (30%) |  |
| Low active |  | 261 (24%) | 101 (18%) | 160 (29%) |  |
| Active |  | 256 (23%) | 133 (24%) | 122 (23%) |  |
| Very active |  | 326 (30%) | 227 (41%) | 98 (18%) |  |
| **Diet type** | 1,100 |  |  |  | 0.002 |
| Omnivorous |  | 1,029 (94%) | 538 (96%) | 491 (91%) |  |
| Vegetarian or vegan |  | 71 (6%) | 20 (4%) | 51 (9%) |  |
| *^1^*Mean ± SE; n (%) | | | | | |
| *^2^*Design-based Wilcoxon rank-sum test; Design-based Kruskal-Wallis rank-sum test; chi-squared test with Rao & Scott's second-order correction | | | | | |
| *^3^*According to the definition of the WHO (12) | | | | | |
| *^4^*According to Gerrior et al. (10) | | | | | |

Supplementary Table 3 Sociodemographic characteristics of the GHGE quintiles. Data are weighted to represent the Bavarian population.
Abbreviations: GHGE greenhouse gas emissions

|  |  | **Quintiles GHGE** | | | | |  |
| --- | --- | --- | --- | --- | --- | --- | --- |
| **Variable** | **Overall**, N = 1100 (100%)*^1^* | **Lowest**, N = 220 (20%)*^1^* | **Low**, N = 221 (20%)*^1^* | **Medium**, N = 219 (20%)*^1^* | **High**, N = 221 (20%)*^1^* | **Highest**, N = 219 (20%)*^1^* | **p‑value***^2^***/ p‑trend***^3^* |
| **Sex** |  |  |  |  |  |  | >0.9 |
| Male | 558 (51%) | 111 (50%) | 102 (46%) | 116 (53%) | 115 (52%) | 114 (52%) |  |
| Female | 542 (49%) | 109 (50%) | 119 (54%) | 102 (47%) | 106 (48%) | 105 (48%) |  |
| **Age (in years)** | 46.7 ± 0.8 | 43.3 ± 1.6 | 44.0 ± 1.7 | 49.0 ± 1.6 | 50.2 ± 1.7 | 47.2 ± 1.9 | 0.011 |
| **Age group** |  |  |  |  |  |  | 0.123 |
| 18-24 | 105 (10%) | 22 (10%) | 28 (13%) | 17 (8%) | 11 (5%) | 26 (12%) |  |
| 25-34 | 202 (18%) | 59 (27%) | 41 (19%) | 28 (13%) | 45 (20%) | 29 (13%) |  |
| 35-50 | 311 (28%) | 63 (28%) | 67 (30%) | 70 (32%) | 43 (19%) | 68 (31%) |  |
| 51-64 | 317 (29%) | 58 (26%) | 65 (30%) | 58 (26%) | 80 (36%) | 57 (26%) |  |
| ≥65 | 165 (15%) | 19 (9%) | 19 (9%) | 46 (21%) | 42 (19%) | 40 (18%) |  |
| **BMI (in kg/m^2^)** | 26.0 ± 0.3 | 24.1 ± 0.4 | 26.5 ± 0.8 | 26.3 ± 0.5 | 26.2 ± 0.5 | 27.1 ± 0.6 | <0.001 |
| **BMI group***^4^* |  |  |  |  |  |  | 0.007 |
| Underweight | 18 (2%) | 5 (2%) | 7 (3%) | 1 (1%) | 1 (0%) | 4 (2%) |  |
| Normal weight | 514 (47%) | 142 (64%) | 101 (46%) | 102 (47%) | 91 (41%) | 79 (36%) |  |
| Pre-obesity | 362 (33%) | 59 (27%) | 62 (28%) | 67 (31%) | 92 (41%) | 82 (37%) |  |
| Obesity | 206 (19%) | 15 (7%) | 51 (23%) | 49 (22%) | 38 (17%) | 53 (24%) |  |
| **Waist circumference (in cm)** | 93.9 ± 0.8 | 89.2 ± 1.4 | 93.9 ± 2.1 | 94.3 ± 1.7 | 95.4 ± 1.6 | 96.9 ± 1.8 | 0.001 |
| **Education** |  |  |  |  |  |  | 0.081 |
| Low | 365 (33%) | 46 (21%) | 69 (31%) | 80 (37%) | 99 (45%) | 71 (32%) |  |
| Middle | 322 (29%) | 64 (29%) | 66 (30%) | 71 (33%) | 54 (24%) | 67 (31%) |  |
| High | 413 (38%) | 111 (50%) | 87 (39%) | 67 (31%) | 68 (31%) | 81 (37%) |  |
| **Equivalized net household income (in Euro/month)** | 2,089.0 ± 75.0 | 2,242.2 ± 180.9 | 1,905.5 ± 132.3 | 1,978.7 ± 159.2 | 2,100.0 ± 167.8 | 2,221.8 ± 187.9 | 0.758 |
| **Civil status** |  |  |  |  |  |  | 0.052 |
| Single | 160 (15%) | 27 (12%) | 34 (15%) | 22 (10%) | 30 (13%) | 47 (21%) |  |
| Unmarried ‑ in a partnership | 193 (18%) | 53 (24%) | 49 (22%) | 35 (16%) | 31 (14%) | 24 (11%) |  |
| Married | 659 (60%) | 131 (60%) | 126 (57%) | 144 (66%) | 138 (62%) | 120 (55%) |  |
| Widowed | 32 (3%) | 0 (0%) | 3 (1%) | 3 (2%) | 9 (4%) | 17 (8%) |  |
| Divorced | 56 (5%) | 9 (4%) | 9 (4%) | 13 (6%) | 14 (6%) | 11 (5%) |  |
| **Living situation** |  |  |  |  |  |  | 0.819 |
| Living alone in a private household | 179 (16%) | 30 (14%) | 30 (14%) | 36 (16%) | 38 (17%) | 45 (21%) |  |
| Living in a private household with family/friends or other persons | 920 (84%) | 191 (86%) | 190 (86%) | 183 (84%) | 182 (83%) | 174 (79%) |  |
| Other | 1 (0%) | 0 (0%) | 1 (0%) | 0 (0%) | 0 (0%) | 0 (0%) |  |
| **Employment** |  |  |  |  |  |  | 0.538 |
| Employed | 702 (64%) | 149 (67%) | 144 (65%) | 139 (63%) | 138 (62%) | 133 (61%) |  |
| Marginally, occasionally or irregularly employed | 29 (3%) | 12 (5%) | 5 (2%) | 3 (1%) | 7 (3%) | 3 (1%) |  |
| In vocational training/apprenticeship/retraining | 26 (2%) | 5 (2%) | 9 (4%) | 5 (2%) | 0 (0%) | 7 (3%) |  |
| Currently not employed: unemployed or job-seeking, on parental leave | 58 (5%) | 12 (6%) | 14 (6%) | 6 (3%) | 10 (5%) | 15 (7%) |  |
| Retired, pensioner, homemaker | 228 (21%) | 34 (15%) | 29 (13%) | 54 (25%) | 56 (25%) | 55 (25%) |  |
| Other (e.g., pupil, student, assisting family member) | 57 (5%) | 9 (4%) | 20 (9%) | 12 (5%) | 10 (5%) | 6 (3%) |  |
| **Smoking** |  |  |  |  |  |  | 0.413 |
| Never | 554 (50%) | 101 (46%) | 133 (60%) | 103 (47%) | 95 (43%) | 122 (56%) |  |
| Currently | 184 (17%) | 36 (17%) | 35 (16%) | 43 (19%) | 43 (20%) | 27 (12%) |  |
| In the past | 362 (33%) | 83 (38%) | 53 (24%) | 73 (33%) | 82 (37%) | 70 (32%) |  |
| **Physical activity group***^5^* |  |  |  |  |  |  | 0.441 |
| Sedentary | 258 (23%) | 63 (29%) | 50 (22%) | 39 (18%) | 49 (22%) | 57 (26%) |  |
| Low active | 261 (24%) | 60 (27%) | 67 (30%) | 42 (19%) | 50 (23%) | 42 (19%) |  |
| Active | 256 (23%) | 49 (22%) | 54 (24%) | 53 (24%) | 49 (22%) | 50 (23%) |  |
| Very active | 326 (30%) | 49 (22%) | 50 (23%) | 85 (39%) | 72 (33%) | 69 (32%) |  |
| **Diet type** |  |  |  |  |  |  | <0.001 |
| Omnivorous | 1,029 (94%) | 180 (82%) | 201 (91%) | 214 (98%) | 218 (99%) | 217 (99%) |  |
| Vegetarian or vegan | 71 (6%) | 40 (18%) | 21 (9%) | 5 (2%) | 3 (1%) | 2 (1%) |  |
| *^1^*n (%); Mean ± SE | | | | | | | |
| *^2^*Chi-squared test with Rao & Scott's second-order correction for categorical variables | | | | | | | |
| *^3^*Design-based generalized linear model with ordered factor levels for metric variables | | | | | | | |
| *^4^*According to the definition of the WHO (12) | | | | | | | |
| *^5^*According to Gerrior et al. (10) | | | | | | | |

Supplementary Table 4 Contribution of food groups to GHGE, stratified by BMI group and energy-adjusted to 2,500 kcal. Data are weighted to represent the Bavarian population.
Abbreviations: GHGE greenhouse gas emissions

|  | **BMI group***^1^* | | | |
| --- | --- | --- | --- | --- |
| **Variable** | **Underweight**, N = 18 (2%) | **Normal weight**, N = 514 (47%) | **Pre-obesity**, N = 362 (33%) | **Obesity**, N = 206 (19%) |
| **Meat, sausages, and meat products** | 22.8% | 24.9% | 36% | 38.7% |
| **Fish and fish products** | 1% | 3.1% | 3.6% | 3% |
| **Eggs** | 1% | 1.3% | 1.1% | 1% |
| **Milk and dairy products** | 19.4% | 21.3% | 18% | 17.5% |
| **Butter** | 15.5% | 9.1% | 8% | 8.5% |
| **Grain products** | 6.1% | 6.5% | 5.8% | 4.9% |
| **Vegetables** | 7.1% | 7.5% | 5.7% | 4.6% |
| **Fruits** | 2.6% | 3.2% | 2.3% | 2% |
| **Legumes, nuts, potatoes, and substitute products** | 2% | 1.8% | 1.3% | 1.3% |
| **Non-alcoholic and alcoholic beverages** | 4.3% | 4.9% | 5.4% | 6.3% |
| **Roasted coffee** | 1.9% | 4.9% | 5.2% | 4.7% |
| **Tea and other infusions** | 13.3% | 6% | 3.6% | 2.7% |
| **Other** | 3.2% | 5.5% | 4.2% | 4.8% |
| *^1^*According to the definition of the WHO (12) | | | | |

Supplementary Table 5 Contribution of food groups to GHGE per day, stratified by BMI group. Data are weighted to represent the Bavarian population.
Abbreviations: GHGE greenhouse gas emissions

|  | **BMI group***^1^* | | | |
| --- | --- | --- | --- | --- |
| **Variable** | **Underweight**, N = 18 (2%) | **Normal weight**, N = 514 (47%) | **Pre-obesity**, N = 362 (33%) | **Obesity**, N = 206 (19%) |
| **Meat, sausages, and meat products** | 25% | 25.2% | 35.5% | 39.8% |
| **Fish and fish products** | 1.2% | 2.9% | 3.3% | 2.9% |
| **Eggs** | 1% | 1.3% | 1.2% | 1% |
| **Milk and dairy products** | 19.9% | 21.3% | 18.8% | 17.2% |
| **Butter** | 15.5% | 9.6% | 8.4% | 8.3% |
| **Grain products** | 5.5% | 6.5% | 5.9% | 4.9% |
| **Vegetables** | 7% | 7.3% | 5.5% | 4.4% |
| **Fruits** | 2.7% | 3.1% | 2.2% | 2% |
| **Legumes, nuts, potatoes, and substitute products** | 1.9% | 1.8% | 1.3% | 1.2% |
| **Non-alcoholic and alcoholic beverages** | 3.7% | 5.1% | 5.5% | 6.7% |
| **Roasted coffee** | 1.4% | 4.9% | 4.8% | 4.5% |
| **Tea and other infusions** | 12.1% | 5.2% | 3.1% | 2.3% |
| **Other** | 3.2% | 5.7% | 4.4% | 4.8% |
| *^1^*According to the definition of the WHO (12) | | | | |

Supplementary Table 6 Food consumption across the GHGE quintiles. Intakes of food and beverage groups are in g/2,500 kcal. P-values for linear trend. Data are weighted to represent the Bavarian population.
Abbreviations: GHGE greenhouse gas emissions

|  |  | **Quintiles GHGE** | | | | |  |
| --- | --- | --- | --- | --- | --- | --- | --- |
| **Variable** | **Overall**, N = 1100 (100%)*^1^* | **Lowest**, N = 220 (20%)*^1^* | **Low**, N = 221 (20%)*^1^* | **Medium**, N = 219 (20%)*^1^* | **High**, N = 221 (20%)*^1^* | **Highest**, N = 219 (20%)*^1^* | **p‑trend***^2^* |
| **Meat** | 73 ± 4 (59) | 29 ± 5 (3) | 48 ± 5 (35) | 63 ± 7 (55) | 86 ± 6 (78) | 141 ± 10 (126) | <0.001 |
| Meat, uncategorized | 9 ± 1 (0) | 1 ± 1 (0) | 10 ± 3 (0) | 4 ± 1 (0) | 14 ± 3 (0) | 17 ± 4 (0) | <0.001 |
| Beef | 14 ± 2 (0) | 1 ± 1 (0) | 3 ± 1 (0) | 9 ± 2 (0) | 19 ± 3 (0) | 39 ± 6 (20) | <0.001 |
| Veal | 1 ± 0 (0) | 0 ± 0 (0) | 0 ± 0 (0) | 2 ± 1 (0) | 0 ± 0 (0) | 4 ± 1 (0) | <0.001 |
| Pork | 20 ± 2 (0) | 12 ± 3 (0) | 20 ± 3 (0) | 14 ± 3 (0) | 25 ± 4 (0) | 31 ± 7 (0) | 0.001 |
| Mutton/Lamb | 1 ± 0 (0) | 0 ± 0 (0) | 0 ± 0 (0) | 0 ± 0 (0) | 1 ± 0 (0) | 2 ± 1 (0) | <0.001 |
| Horse-, goat-, rabbit meat, and game mammals | 1 ± 0 (0) | 0 ± 0 (0) | 0 ± 0 (0) | 0 ± 0 (0) | 0 ± 0 (0) | 2 ± 1 (0) | <0.001 |
| Poultry and game poultry | 27 ± 3 (0) | 14 ± 4 (0) | 14 ± 3 (0) | 33 ± 7 (0) | 28 ± 5 (0) | 44 ± 9 (0) | <0.001 |
| Variety meat and offal | 0 ± 0 (0) | 0 ± 0 (0) | 0 ± 0 (0) | 0 ± 0 (0) | 0 ± 0 (0) | 2 ± 1 (0) | <0.001 |
| **Meat and sausage products** | 53 ± 3 (39) | 30 ± 6 (8) | 49 ± 5 (43) | 58 ± 5 (47) | 61 ± 7 (51) | 69 ± 7 (63) | <0.001 |
| Sausage and sausage products | 43 ± 3 (26) | 24 ± 5 (0) | 39 ± 5 (26) | 48 ± 5 (35) | 51 ± 7 (30) | 54 ± 7 (35) | <0.001 |
| Ham and cured meat | 10 ± 1 (0) | 6 ± 1 (0) | 9 ± 2 (0) | 10 ± 2 (0) | 9 ± 2 (0) | 15 ± 3 (5) | 0.003 |
| Canned meat | 0 ± 0 (0) | 0 ± 0 (0) | 1 ± 1 (0) | 0 ± 0 (0) | 0 ± 0 (0) | 0 ± 0 (0) | <0.001 |
| **Fish and fish products** | 24 ± 2 (0) | 16 ± 3 (0) | 17 ± 4 (0) | 24 ± 5 (0) | 20 ± 4 (0) | 42 ± 6 (0) | <0.001 |
| Fish, fresh and frozen | 13 ± 1 (0) | 7 ± 2 (0) | 10 ± 3 (0) | 10 ± 3 (0) | 10 ± 2 (0) | 26 ± 5 (0) | 0.004 |
| Canned fish | 10 ± 1 (0) | 6 ± 2 (0) | 5 ± 2 (0) | 13 ± 5 (0) | 8 ± 2 (0) | 16 ± 4 (0) | 0.010 |
| Other fish products | 1 ± 0 (0) | 3 ± 1 (0) | 1 ± 1 (0) | 1 ± 0 (0) | 2 ± 1 (0) | 0 ± 0 (0) | 0.126 |
| **Eggs** | 26 ± 2 (13) | 23 ± 3 (15) | 23 ± 3 (15) | 26 ± 3 (14) | 32 ± 6 (17) | 24 ± 5 (5) | 0.412 |
| **Milk and dairy products** | 217 ± 8 (181) | 185 ± 15 (155) | 226 ± 17 (208) | 191 ± 16 (163) | 264 ± 22 (227) | 220 ± 18 (206) | 0.049 |
| Milk | 91 ± 6 (37) | 68 ± 10 (30) | 110 ± 16 (61) | 72 ± 9 (31) | 114 ± 17 (41) | 87 ± 13 (47) | 0.256 |
| Cream | 2 ± 0 (0) | 2 ± 1 (0) | 3 ± 1 (0) | 1 ± 0 (0) | 2 ± 1 (0) | 2 ± 1 (0) | 0.763 |
| Cream cheese, quark | 18 ± 3 (0) | 12 ± 3 (0) | 17 ± 6 (0) | 14 ± 3 (0) | 24 ± 8 (0) | 24 ± 6 (0) | 0.056 |
| Fermented dairy products | 52 ± 4 (0) | 58 ± 11 (0) | 38 ± 6 (0) | 45 ± 10 (0) | 67 ± 10 (0) | 53 ± 9 (0) | 0.477 |
| Other milk-based and dairy products | 8 ± 2 (0) | 3 ± 1 (0) | 12 ± 4 (0) | 9 ± 8 (0) | 7 ± 4 (0) | 7 ± 4 (0) | 0.456 |
| Cheese | 47 ± 2 (36) | 41 ± 4 (31) | 46 ± 4 (35) | 50 ± 5 (39) | 48 ± 4 (38) | 47 ± 5 (37) | 0.351 |
| **Butter** | 12 ± 1 (8) | 8 ± 1 (5) | 9 ± 1 (5) | 14 ± 2 (11) | 14 ± 1 (11) | 14 ± 2 (10) | <0.001 |
| **Cooking oils and fats (excluding butter)** | 14 ± 1 (11) | 15 ± 1 (11) | 14 ± 1 (13) | 15 ± 2 (10) | 15 ± 2 (11) | 13 ± 1 (11) | 0.685 |
| Margarine | 2 ± 0 (0) | 2 ± 0 (0) | 1 ± 0 (0) | 2 ± 0 (0) | 3 ± 1 (0) | 1 ± 0 (0) | 0.711 |
| Plant-based fats and oils | 7 ± 0 (5) | 8 ± 1 (4) | 8 ± 1 (5) | 6 ± 1 (4) | 6 ± 1 (4) | 8 ± 1 (6) | 0.603 |
| Mayonnaise and other fat-based products | 4 ± 0 (0) | 4 ± 1 (0) | 5 ± 1 (0) | 6 ± 2 (0) | 5 ± 1 (0) | 3 ± 1 (0) | 0.566 |
| Animal-based fats and oils | 0 ± 0 (0) | 0 ± 0 (0) | 0 ± 0 (0) | 0 ± 0 (0) | 0 ± 0 (0) | 0 ± 0 (0) | 0.207 |
| Cooking fats and oils, uncategorized | 1 ± 0 (0) | 1 ± 0 (0) | 0 ± 0 (0) | 1 ± 0 (0) | 1 ± 0 (0) | 1 ± 0 (0) | 0.583 |
| **Bread and bakery products** | 157 ± 4 (151) | 173 ± 10 (170) | 154 ± 8 (143) | 167 ± 9 (168) | 147 ± 9 (145) | 144 ± 8 (147) | 0.021 |
| White bread, crispbread, bread rolls | 62 ± 3 (51) | 68 ± 6 (63) | 57 ± 6 (45) | 71 ± 8 (62) | 53 ± 6 (44) | 59 ± 7 (41) | 0.256 |
| Other bread | 32 ± 2 (22) | 28 ± 4 (0) | 29 ± 3 (22) | 34 ± 4 (28) | 38 ± 4 (28) | 33 ± 4 (22) | 0.210 |
| Baked goods and pastries | 63 ± 3 (46) | 76 ± 10 (57) | 69 ± 6 (56) | 61 ± 7 (40) | 56 ± 6 (46) | 53 ± 7 (30) | 0.020 |
| **Grain-based staple foods** | 136 ± 6 (102) | 162 ± 18 (135) | 166 ± 14 (146) | 130 ± 14 (100) | 115 ± 10 (101) | 105 ± 12 (78) | <0.001 |
| Flour | 6 ± 1 (0) | 12 ± 6 (0) | 5 ± 1 (0) | 3 ± 1 (0) | 6 ± 2 (0) | 3 ± 1 (0) | 0.017 |
| Rice | 30 ± 4 (0) | 35 ± 13 (0) | 35 ± 10 (0) | 33 ± 7 (0) | 21 ± 4 (0) | 27 ± 6 (0) | 0.292 |
| Grains (excluding rice) | 13 ± 2 (0) | 24 ± 6 (0) | 11 ± 4 (0) | 11 ± 3 (0) | 10 ± 2 (0) | 8 ± 2 (0) | 0.005 |
| Other grain products | 6 ± 1 (0) | 8 ± 1 (0) | 5 ± 1 (0) | 6 ± 2 (0) | 5 ± 2 (0) | 4 ± 1 (0) | 0.117 |
| Pasta products | 81 ± 5 (53) | 84 ± 13 (17) | 110 ± 14 (71) | 77 ± 11 (35) | 73 ± 9 (56) | 63 ± 10 (0) | 0.042 |
| **Wholegrain products** | 24 ± 1 (8) | 31 ± 4 (20) | 22 ± 3 (9) | 21 ± 3 (0) | 21 ± 3 (0) | 23 ± 3 (8) | 0.103 |
| Wholegrain pasta products | 1 ± 1 (0) | 4 ± 3 (0) | 1 ± 1 (0) | 0 ± 0 (0) | 0 ± 0 (0) | 1 ± 1 (0) | 0.035 |
| Muesli | 4 ± 1 (0) | 6 ± 2 (0) | 4 ± 1 (0) | 3 ± 1 (0) | 5 ± 2 (0) | 3 ± 1 (0) | 0.145 |
| Wholegrain bread and bread rolls | 18 ± 1 (0) | 21 ± 4 (0) | 17 ± 3 (0) | 18 ± 3 (0) | 16 ± 3 (0) | 19 ± 2 (0) | 0.585 |
| **Potatoes and potato products** | 89 ± 5 (66) | 96 ± 15 (48) | 75 ± 7 (60) | 85 ± 9 (71) | 101 ± 10 (77) | 91 ± 9 (73) | 0.637 |
| Potatoes, fresh | 83 ± 5 (61) | 88 ± 15 (48) | 72 ± 7 (60) | 81 ± 9 (64) | 93 ± 10 (65) | 84 ± 8 (71) | 0.689 |
| Potato products | 6 ± 2 (0) | 8 ± 5 (0) | 3 ± 1 (0) | 4 ± 2 (0) | 8 ± 3 (0) | 7 ± 3 (0) | 0.749 |
| **Vegetables** | 232 ± 8 (193) | 228 ± 15 (215) | 231 ± 19 (183) | 194 ± 15 (160) | 234 ± 17 (193) | 273 ± 19 (227) | 0.088 |
| Vegetables, uncategorized | 19 ± 2 (0) | 18 ± 4 (0) | 18 ± 4 (0) | 13 ± 3 (0) | 20 ± 5 (0) | 24 ± 4 (0) | 0.254 |
| Salad vegetables | 26 ± 2 (14) | 27 ± 4 (17) | 21 ± 3 (5) | 26 ± 4 (12) | 29 ± 4 (20) | 28 ± 4 (11) | 0.359 |
| Leafy and stalk vegetables | 6 ± 1 (0) | 10 ± 4 (0) | 10 ± 3 (0) | 4 ± 1 (0) | 2 ± 1 (0) | 4 ± 2 (0) | 0.013 |
| Cabbage vegetables | 22 ± 2 (0) | 21 ± 4 (0) | 20 ± 4 (0) | 24 ± 6 (0) | 20 ± 4 (0) | 25 ± 5 (1) | 0.519 |
| Sprout and leek vegetables | 23 ± 1 (13) | 25 ± 4 (17) | 22 ± 3 (12) | 18 ± 2 (12) | 19 ± 2 (13) | 32 ± 5 (15) | 0.381 |
| Fruit vegetables | 105 ± 5 (73) | 93 ± 10 (68) | 100 ± 11 (67) | 82 ± 9 (38) | 120 ± 13 (93) | 131 ± 13 (96) | 0.008 |
| Root and tuber vegetables | 21 ± 2 (5) | 21 ± 5 (9) | 31 ± 7 (9) | 18 ± 3 (3) | 15 ± 2 (4) | 20 ± 4 (5) | 0.214 |
| Oil fruits | 1 ± 0 (0) | 1 ± 0 (0) | 1 ± 0 (0) | 1 ± 1 (0) | 0 ± 0 (0) | 2 ± 1 (0) | 0.152 |
| Mushrooms | 5 ± 1 (0) | 7 ± 3 (0) | 4 ± 1 (0) | 4 ± 1 (0) | 4 ± 1 (0) | 4 ± 1 (0) | 0.326 |
| Vegetable products | 4 ± 1 (0) | 5 ± 1 (0) | 5 ± 2 (0) | 4 ± 1 (0) | 3 ± 1 (0) | 1 ± 1 (0) | 0.008 |
| **Legumes and pulses** | 16 ± 3 (0) | 28 ± 9 (0) | 15 ± 5 (0) | 9 ± 3 (0) | 18 ± 7 (0) | 11 ± 2 (0) | 0.064 |
| **Fruits** | 163 ± 9 (120) | 190 ± 24 (124) | 146 ± 27 (73) | 157 ± 18 (92) | 146 ± 13 (121) | 176 ± 18 (147) | 0.403 |
| Fruits, uncategorized | 13 ± 3 (0) | 22 ± 12 (0) | 11 ± 5 (0) | 10 ± 4 (0) | 14 ± 4 (0) | 9 ± 4 (0) | 0.695 |
| Pome fruits | 51 ± 4 (0) | 67 ± 10 (24) | 38 ± 6 (0) | 49 ± 8 (0) | 44 ± 6 (0) | 56 ± 10 (0) | 0.290 |
| Stone fruits | 12 ± 2 (0) | 13 ± 6 (0) | 8 ± 2 (0) | 17 ± 8 (0) | 6 ± 2 (0) | 18 ± 6 (0) | 0. 782 |
| Berries | 16 ± 2 (0) | 13 ± 4 (0) | 15 ± 4 (0) | 17 ± 4 (0) | 18 ± 4 (0) | 17 ± 4 (0) | 0.396 |
| Wild fruits | 0 ± 0 (0) | 0 ± 0 (0) | 0 ± 0 (0) | 0 ± 0 (0) | 0 ± 0 (0) | 0 ± 0 (0) | *NA* |
| Raisins | 0 ± 0 (0) | 1 ± 1 (0) | 0 ± 0 (0) | 0 ± 0 (0) | 0 ± 0 (0) | 0 ± 0 (0) | 0.008 |
| Tropical fruits | 51 ± 6 (0) | 50 ± 9 (19) | 57 ± 26 (0) | 50 ± 8 (0) | 42 ± 7 (0) | 54 ± 12 (0) | 0.845 |
| Citrus fruits | 19 ± 2 (0) | 23 ± 6 (0) | 15 ± 4 (0) | 14 ± 4 (0) | 20 ± 5 (0) | 20 ± 5 (0) | 0.987 |
| Canned fruits | 1 ± 0 (0) | 0 ± 0 (0) | 2 ± 1 (0) | 0 ± 0 (0) | 2 ± 2 (0) | 1 ± 0 (0) | 0.569 |
| **Nuts, kernels, and seeds** | 9 ± 1 (0) | 13 ± 2 (0) | 11 ± 2 (0) | 7 ± 2 (0) | 7 ± 1 (0) | 7 ± 2 (0) | 0.007 |
| **Sugars and sweeteners** | 2 ± 0 (0) | 2 ± 1 (0) | 2 ± 0 (0) | 2 ± 0 (0) | 2 ± 1 (0) | 2 ± 1 (0) | 0.830 |
| Sugars | 2 ± 0 (0) | 2 ± 1 (0) | 1 ± 0 (0) | 1 ± 0 (0) | 2 ± 1 (0) | 2 ± 1 (0) | 0.981 |
| Sweeteners | 0 ± 0 (0) | 0 ± 0 (0) | 0 ± 0 (0) | 0 ± 0 (0) | 0 ± 0 (0) | 0 ± 0 (0) | 0.076 |
| **Marmalade, jam, and jelly** | 7 ± 1 (0) | 8 ± 2 (0) | 6 ± 1 (0) | 9 ± 2 (0) | 6 ± 1 (0) | 6 ± 1 (0) | 0.444 |
| **Sweets** | 24 ± 2 (12) | 24 ± 3 (12) | 28 ± 3 (19) | 23 ± 3 (14) | 27 ± 4 (13) | 20 ± 3 (3) | 0.349 |
| Cocoa and cocoa drink powders | 0 ± 0 (0) | 0 ± 0 (0) | 0 ± 0 (0) | 0 ± 0 (0) | 0 ± 0 (0) | 0 ± 0 (0) | 0.914 |
| Chocolates and chocolate products | 9 ± 1 (0) | 8 ± 2 (0) | 10 ± 2 (0) | 12 ± 2 (0) | 12 ± 2 (0) | 5 ± 1 (0) | 0.119 |
| Confectionery and other sweets | 2 ± 0 (0) | 3 ± 1 (0) | 3 ± 1 (0) | 2 ± 2 (0) | 1 ± 1 (0) | 2 ± 1 (0) | 0.389 |
| Ice cream | 8 ± 1 (0) | 9 ± 3 (0) | 9 ± 2 (0) | 5 ± 1 (0) | 10 ± 3 (0) | 9 ± 3 (0) | 0.755 |
| Honey and sweet spreads | 4 ± 0 (0) | 5 ± 1 (0) | 5 ± 1 (0) | 4 ± 1 (0) | 3 ± 1 (0) | 3 ± 1 (0) | 0.199 |
| **Seasonings and other ingredients** | 25 ± 1 (20) | 24 ± 2 (20) | 21 ± 2 (17) | 26 ± 4 (20) | 27 ± 2 (23) | 27 ± 3 (20) | 0.174 |
| **Non-alcoholic beverages** | 2,462 ± 77 (2,225) | 2,542 ± 187 (2,392) | 2,227 ± 152 (1,961) | 2,343 ± 135 (2,104) | 2,406 ± 175 (2,161) | 2,794 ± 189 (2,451) | 0.234 |
| Fruit and vegetable juices | 41 ± 5 (0) | 40 ± 7 (0) | 40 ± 14 (0) | 47 ± 12 (0) | 51 ± 14 (0) | 28 ± 8 (0) | 0.592 |
| Table water | 2,130 ± 80 (1,860) | 2,264 ± 200 (1,923) | 1,878 ± 144 (1,618) | 1,954 ± 145 (1,729) | 2,108 ± 182 (1,764) | 2,447 ± 194 (2,122) | 0.304 |
| Juice spritzer | 78 ± 11 (0) | 62 ± 22 (0) | 98 ± 29 (0) | 84 ± 27 (0) | 54 ± 14 (0) | 94 ± 26 (0) | 0.796 |
| Sodas and lemonades | 141 ± 15 (0) | 88 ± 21 (0) | 153 ± 28 (0) | 188 ± 42 (0) | 130 ± 31 (0) | 144 ± 41 (0) | 0.313 |
| Other non-alcoholic beverages | 36 ± 6 (0) | 55 ± 14 (0) | 18 ± 5 (0) | 26 ± 8 (0) | 34 ± 12 (0) | 45 ± 21 (0) | 0.837 |
| Coffee substitutes | 37 ± 9 (0) | 34 ± 11 (0) | 40 ± 26 (0) | 45 ± 29 (0) | 29 ± 7 (0) | 36 ± 13 (0) | 0.861 |
| **Alcoholic beverages** | 220 ± 17 (0) | 190 ± 35 (0) | 232 ± 34 (70) | 297 ± 53 (105) | 192 ± 34 (0) | 189 ± 34 (0) | 0.724 |
| Spirits | 1 ± 0 (0) | 1 ± 0 (0) | 1 ± 1 (0) | 0 ± 0 (0) | 0 ± 0 (0) | 1 ± 0 (0) | 0.055 |
| Beer | 167 ± 16 (0) | 154 ± 34 (0) | 166 ± 31 (0) | 236 ± 48 (0) | 138 ± 33 (0) | 142 ± 33 (0) | 0.626 |
| Liqueurs and cocktails | 3 ± 1 (0) | 6 ± 3 (0) | 3 ± 2 (0) | 3 ± 2 (0) | 2 ± 1 (0) | 3 ± 2 (0) | 0.362 |
| Wine and sparkling wine | 49 ± 5 (0) | 29 ± 6 (0) | 62 ± 11 (0) | 58 ± 12 (0) | 51 ± 11 (0) | 43 ± 8 (0) | 0.365 |
| **Roasted coffee** | 429 ± 19 (372) | 346 ± 33 (316) | 353 ± 31 (352) | 426 ± 40 (386) | 526 ± 48 (437) | 494 ± 51 (378) | <0.001 |
| **Tea and other infusions** | 374 ± 32 (0) | 202 ± 38 (0) | 222 ± 36 (0) | 414 ± 101 (0) | 360 ± 59 (0) | 676 ± 92 (276) | <0.001 |
| Tea (real) | 141 ± 17 (0) | 104 ± 32 (0) | 58 ± 14 (0) | 78 ± 18 (0) | 139 ± 35 (0) | 328 ± 61 (0) | <0.001 |
| Fruit and herbal tea | 233 ± 27 (0) | 98 ± 22 (0) | 165 ± 31 (0) | 336 ± 101 (0) | 221 ± 49 (0) | 348 ± 57 (0) | <0.001 |
| **Soups and sauces** | 52 ± 3 (29) | 56 ± 7 (36) | 67 ± 8 (45) | 50 ± 7 (24) | 51 ± 9 (20) | 35 ± 5 (17) | 0.005 |
| **Substitute products** | 21 ± 3 (0) | 52 ± 12 (0) | 22 ± 6 (0) | 10 ± 3 (0) | 10 ± 4 (0) | 9 ± 3 (0) | <0.001 |
| Milk substitutes | 16 ± 3 (0) | 38 ± 11 (0) | 20 ± 6 (0) | 9 ± 3 (0) | 9 ± 4 (0) | 6 ± 3 (0) | <0.001 |
| Meat substitutes | 4 ± 1 (0) | 14 ± 4 (0) | 3 ± 1 (0) | 1 ± 1 (0) | 1 ± 1 (0) | 3 ± 2 (0) | 0.019 |
| **Desserts and other sweet dishes** | 19 ± 2 (0) | 23 ± 6 (0) | 20 ± 5 (0) | 14 ± 3 (0) | 20 ± 4 (0) | 17 ± 4 (0) | 0.518 |
| *^1^*Mean ± SE (Median) | | | | | | | |
| *^2^*Design-based generalized linear model with ordered factor levels | | | | | | | |

Supplementary Table 7 Sampling exhaustion and reasons for non-response and dropout for the home visits

|  | **N** | **%** | **%** |
| --- | --- | --- | --- |
| **Gross sample size** | **7,449** | **100.0** |  |
| Quality-neutral dropouts (QNDs): | 1,679 | 22.5 |  |
| Adress-related QNDs: | 582 | 7.8 | 100.0 |
| Non-existent address | 53 | 0.7 | 9.1 |
| Moved – within Germany | 87 | 1.2 | 14.9 |
| Moved – abroad | 46 | 0.6 | 7.9 |
| Moved – unknown | 396 | 5.3 | 68.0 |
| Other QNDs: | 1,097 | 14.7 | 100.0 |
| Deceased | 10 | 0.1 | 0.9 |
| Not living in a private household | 28 | 0.4 | 2.6 |
| Insufficient German | 341 | 4.6 | 31.1 |
| In quarantine (COVID-19) | 718 | 9.6 | 65.4 |
| **Cleaned gross sample size** | **5,770** | **77.5** | **100.0** |
| Other dropouts: | 4,267 |  | 74.0 |
| Limit in sample point reached | 284 |  | 4.9 |
| Target person was not reached in the household | 514 |  | 8.9 |
| No appointment could be scheduled | 369 |  | 6.4 |
| Refused to participate or cancelled participation via hotline | 3,056 |  | 53.0 |
| Acutely or chronically ill | 44 |  | 0.8 |
| **Final net sample size - house visits (CAPI/CASI)** | **1,503** |  | **26.0** |

Supplementary Table 8 Sampling exhaustion and exclusions for the dietary assessment

|  | **N** | **%** | **%** |
| --- | --- | --- | --- |
| **Final net sample size - house visits** | **1,503** | **100.0** |  |
| 1x 24-h recall | 91 | 6.1 |  |
| 2x 24-h recalls | 165 | 11.0 |  |
| 3x 24-h recalls | 983 | 65.4 |  |
| No nutritional data | 264 | 17.6 |  |
| **Net sample size (≥2x 24-h recalls): dietary assessment** | **1,148** | **76.4** | **100.0** |
| Underreporters (EI/BMR<0.6) | 48 | 3.2 | 4.2 |
| **Final net sample size - dietary assessment (CATI)** | **1,100** | **73.2** | **95.8** |

# Supplementary Figures


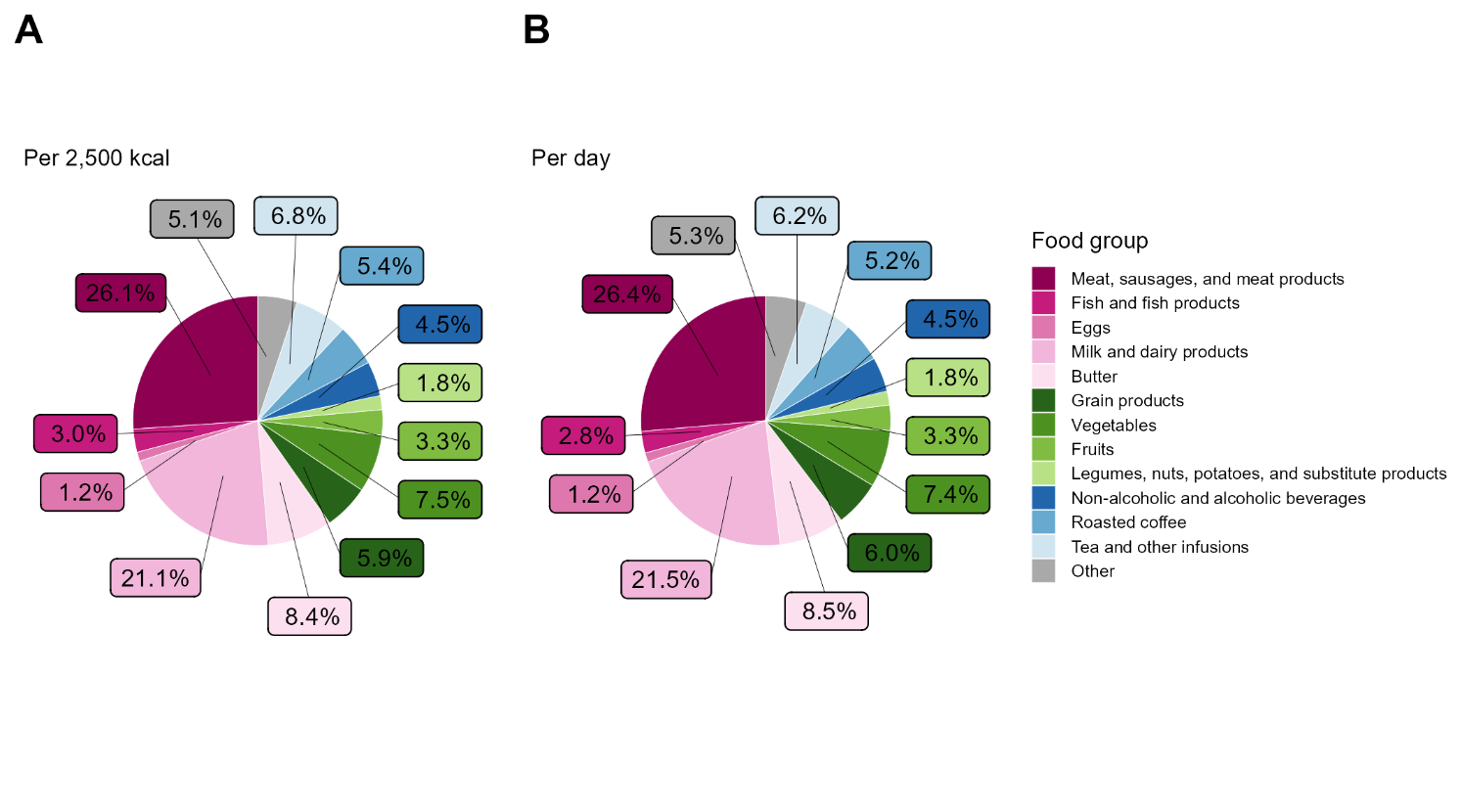


Supplementary Figure 1 Contribution of food groups to GHGE for females (N = 542) (A) per 2,500 kcal and (B) per day. Data are weighted to represent the Bavarian population.
Abbreviations: GHGE greenhouse gas emissions


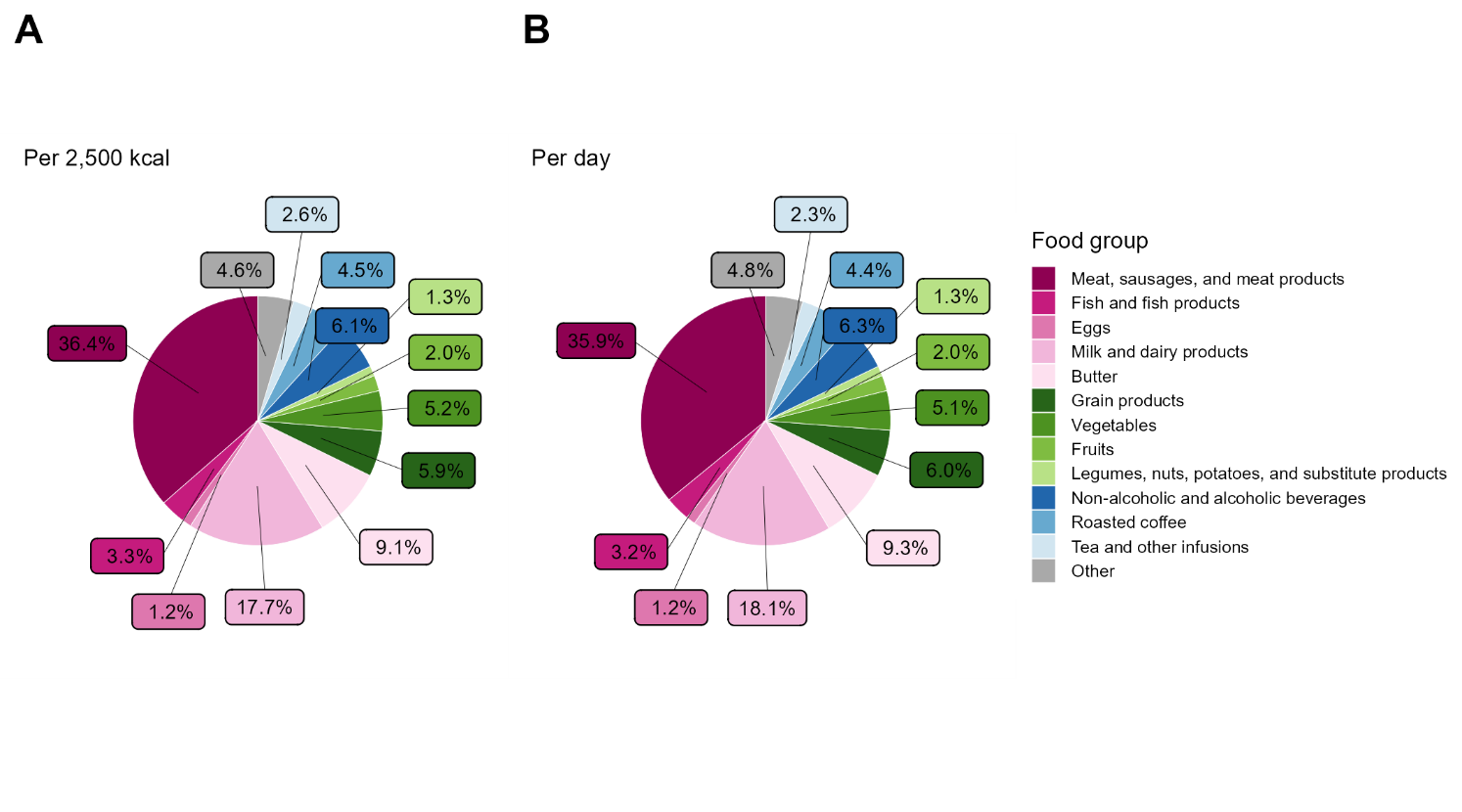


Supplementary Figure 2 Contribution of food groups to GHGE for males (N = 558) (A) per 2,500 kcal and (B) per day. Data are weighted to represent the Bavarian population.
Abbreviations: GHGE greenhouse gas emissions

1. Barone B, Nogueira RM, Guimarães K, Behrens JH. Sustainable Diet from the Urban Brazilian Consumer Perspective. *Food Res Int* (2019) 124:206-12. Epub 20180518. doi: 10.1016/j.foodres.2018.05.027.

2. Tobler C, Visschers VHM, Siegrist M. Eating Green. Consumers’ Willingness to Adopt Ecological Food Consumption Behaviors. *Appetite* (2011) 57(3):674-82. doi: <https://doi.org/10.1016/j.appet.2011.08.010>.

3. Weller KE, Greene GW, Redding CA, Paiva AL, Lofgren I, Nash JT, et al. Development and Validation of Green Eating Behaviors, Stage of Change, Decisional Balance, and Self-Efficacy Scales in College Students. *J Nutr Educ Behav* (2014) 46(5):324-33. Epub 20140305. doi: 10.1016/j.jneb.2014.01.002.

4. Wahl DR, Villinger K, Blumenschein M, König LM, Ziesemer K, Sproesser G, et al. Why We Eat What We Eat: Assessing Dispositional and in-the-Moment Eating Motives by Using Ecological Momentary Assessment. *JMIR Mhealth Uhealth* (2020) 8(1):e13191. Epub 20200107. doi: 10.2196/13191.

5. Renner B, Sproesser G, Strohbach S, Schupp HT. Why We Eat What We Eat. The Eating Motivation Survey (Tems). *Appetite* (2012) 59(1):117-28. Epub 20120419. doi: 10.1016/j.appet.2012.04.004.

6. Bush K, Kivlahan DR, McDonell MB, Fihn SD, Bradley KA. The Audit Alcohol Consumption Questions (Audit-C): An Effective Brief Screening Test for Problem Drinking. Ambulatory Care Quality Improvement Project (Acquip). Alcohol Use Disorders Identification Test. *Arch Intern Med* (1998) 158(16):1789-95. doi: 10.1001/archinte.158.16.1789.

7. Selim AJ, Rogers W, Fleishman JA, Qian SX, Fincke BG, Rothendler JA, et al. Updated U.S. Population Standard for the Veterans Rand 12-Item Health Survey (Vr-12). *Qual Life Res* (2009) 18(1):43-52. Epub 2008/12/04. doi: 10.1007/s11136-008-9418-2.

8. Kazis LE, Miller DR, Clark JA, Skinner KM, Lee A, Ren XS, et al. Improving the Response Choices on the Veterans Sf-36 Health Survey Role Functioning Scales: Results from the Veterans Health Study. *J Ambul Care Manage* (2004) 27(3):263-80. Epub 2004/08/04. doi: 10.1097/00004479-200407000-00010.

9. Finger JD, Tafforeau J, Gisle L, Oja L, Ziese T, Thelen J, et al. Development of the European Health Interview Survey - Physical Activity Questionnaire (Ehis-Paq) to Monitor Physical Activity in the European Union. *Arch Public Health* (2015) 73:59. Epub 20151202. doi: 10.1186/s13690-015-0110-z.

10. Gerrior S, Juan W, Basiotis P. An Easy Approach to Calculating Estimated Energy Requirements. *Prev Chronic Dis* (2006) 3(4):A129. Epub 20060915.

11. Nieters A, Weber S, Elgizouli M. Screening Score to Identify People Prone to Respiratory Tract Infections in the Community. *Int J Respir Med 2017; 2 (1): 6-13 7 Int J Respir Med 2017 Volume 2 Issue* (2017) 1.

12. World Health Organization. Obesity and Overweight (2024) [cited 2024 04.11.2024]. Available from: <https://www.who.int/news-room/fact-sheets/detail/obesity-and-overweight>.
